# XCVATR: detection and characterization of variant impact on the Embeddings of single -cell and bulk RNA-sequencing samples

**DOI:** 10.1186/s12864-022-09004-7

**Published:** 2022-12-20

**Authors:** Arif Harmanci, Akdes Serin Harmanci, Tiemo J. Klisch, Akash J. Patel

**Affiliations:** 1 University of Texas Health Science Center, School of Biomedical Informatics, Center for Secure Artificial intelligence For hEalthcare (SAFE), Center for Precision Health, Houston, USA; 2 Department of Neurosurgery, Baylor College of Medicine, Houston, TX 77030 USA; 3 Jan and Dan Duncan Neurological Research Institute, Texas Children’s Hospital, Houston, TX 77030 USA; 4 Department of Molecular and Human Genetics, Baylor College of Medicine, Houston, TX 77030 USA; 5 Department of Otolaryngology – Head and Neck Surgery, Baylor College of Medicine, Houston, TX 77030 USA

**Keywords:** Single cell RNA-sequencing, Embedding, Genetic variation

## Abstract

**Background:**

RNA-sequencing has become a standard tool for analyzing gene activity in bulk samples and at the single-cell level. By increasing sample sizes and cell counts, this technique can uncover substantial information about cellular transcriptional states. Beyond quantification of gene expression, RNA-seq can be used for detecting variants, including single nucleotide polymorphisms, small insertions/deletions, and larger variants, such as copy number variants. Notably, joint analysis of variants with cellular transcriptional states may provide insights into the impact of mutations, especially for complex and heterogeneous samples. However, this analysis is often challenging due to a prohibitively high number of variants and cells, which are difficult to summarize and visualize. Further, there is a dearth of methods that assess and summarize the association between detected variants and cellular transcriptional states.

**Results:**

Here, we introduce XCVATR (e**X**pressed **C**lusters of **V**ariant **A**lleles in **T**ranscriptome p**R**ofiles), a method that identifies variants and detects local enrichment of expressed variants within embedding of samples and cells in single-cell and bulk RNA-seq datasets. XCVATR visualizes local “clumps” of small and large-scale variants and searches for patterns of association between each variant and cellular states, as described by the coordinates of cell embedding, which can be computed independently using any type of distance metrics, such as principal component analysis or t-distributed stochastic neighbor embedding. Through simulations and analysis of real datasets, we demonstrate that XCVATR can detect enrichment of expressed variants and provide insight into the transcriptional states of cells and samples. We next sequenced 2 new single cell RNA-seq tumor samples and applied XCVATR. XCVATR revealed subtle differences in CNV impact on tumors.

**Conclusions:**

XCVATR is publicly available to download from https://github.com/harmancilab/XCVATR.

**Supplementary Information:**

The online version contains supplementary material available at 10.1186/s12864-022-09004-7.

## Background

Gene expression profiling generates large datasets that contain information about the activity levels of all genes in the transcriptome for a large number of samples. Analysis of these complex and high-dimensional data can uncover hidden expression patterns for driver genes, such as disease markers [1, 2], delineate the transcriptional architecture of disease pathophysiology [3, 4], and help to formulate new hypotheses [5]. RNA-sequencing (RNA-seq), wherein cDNA generated from isolated RNA is sequenced and quantified to estimate of gene expression levels, is the standard approach for profiling gene expression in large samples [6]. Unlike gene expression arrays, which only provide estimates of gene expression levels, RNA-seq can also determine allele-specific expression, facilitate expression quantitative trait locus (eQTL) mapping [7–9], detect small polymorphisms [10], and large copy number variants (CNVs) [11], and elucidate transcriptional dynamics [12, 13]. The decreasing cost of sequencing has facilitated analysis of very large sample sizes that include thousands of samples [14] and hundreds of tissues [15, 16]. Further, advances in single-cell sequencing technologies [17–19] have allowed gene expression profiling at the single-cell level for thousands of cells, encompassing hundreds of cell types and cellular states. Consequently, the amount of information that must be summarized and interpreted is increasing at a challenging pace.

One of the main difficulties researchers face when analyzing large datasets is efficiently summarizing the massive quantities of biological information. To analyze thousands of samples or cells in a meaningful manner [20], it is first necessary to decrease dimensionality of the data by embedding the cells with methods such as principal component analysis (PCA) or t-distributed stochastic neighbor embedding (t-SNE). Embedding enables the transcriptomic state of cells to be summarized and puts them in a simple perspective so that they can be clustered [21] for differential expression analysis [22], cell-type assignment [23], and integration with other datasets across multiple modalities [24]. Critically, while numerous embedding techniques have been proposed [25], they have mostly been used for visualization purposes only. Thus, further work is needed to fully utilize the embedding space in downstream analyses.

Here, we propose a novel method for the detection, integration, and visualization of genetic variation within the embedding space from single-cell and bulk RNA-seq datasets. Motivation for developing this method stems from our observation that cells with similar mutations generally cluster together in “clumps”, and analyzing such clumps can yield interesting insights and facilitate generation of novel hypotheses. For example, a driver mutation might induce a specific transcriptional state, which causes cells harboring the mutation to cluster in the embedding coordinates. The most consequential variants are large-scale CNVs that show clear clumping patterns in t-SNE and PCA embeddings of gene expression analyses. Our approach, named XCVATR (e**X**pressed **C**lusters of **V**ariant **A**lleles in **T**ranscriptome p**R**ofiles), is a flexible and integrated framework for detecting, filtering, and analyzing mutations, so as to determine their association with the distances that are defined by the cell-embedding techniques (i.e.*,* spatial enrichment of mutations on the embedding space).

XCVATR is different from clustering methods that use variant calls to cluster cells [26, 27] in two main ways. First, XCVATR maps the variant allele frequencies (AFs) on an existing embedding and detects local patterns of enrichment for the expressed alleles (i.e., spatial correlation between the expressed variant [28] AFs and embedding coordinates). This is distinct from clustering methods that define the distance metric using the variants themselves. Second, unlike clustering algorithms that aim to identify cell clusters to optimize global clustering of the cells, XCVATR identifies local patterns. In addition, XCVATR is a self-contained framework for the detection, annotation, and filtering of small variants, as well as for the detection and visualization of associations between variant AFs and the embedding space. Thus, there is no dependency on other methods, and the parameters for variant calling and filtering can be explicitly controlled.

A major component of XCVATR is the embedding that is used to summarize cellular transcriptomic states and define cell–cell distances. XCVATR expects the embedding to preserve locality information, such that cells close to one another in the embedding space are biologically similar. This is a reasonable expectation for popular dimensionality reduction techniques, such as PCA, t-SNE, and uniform manifold approximation and projection (UMAP). Among these, t-SNE and UMAP probabilistically preserve locality (i.e.*,* there is a random component in the embedding). Here, we demonstrate that locality information is fairly well-preserved, even with the presence of randomness in the embedding. We note, however, that the opposite statement does not have to hold. That is, we do not expect all biologically similar cells to map close to each other in the embedding space, as this strict requirement would require the embedding to preserve biological information almost exactly [29]. Furthermore, it is not necessary to analyze the association of variants with embedding coordinates and geometry. Overall, XCVATR combines various components of general RNA-seq analysis into one flexible package for identifying, filtering, and visualizing variants at different levels (e.g., at variant level, gene level, and large scale events), while also analyzing their spatial distributions. Thus, we expect that this will be a useful tool for detecting genetic variants and assessing their biological impact in single-cell and bulk RNA-seq datasets.

## Results

We first overview the XCVATR algorithm then we present the main results.

### Overview of XCVATR algorithm

**Fig. 1:**
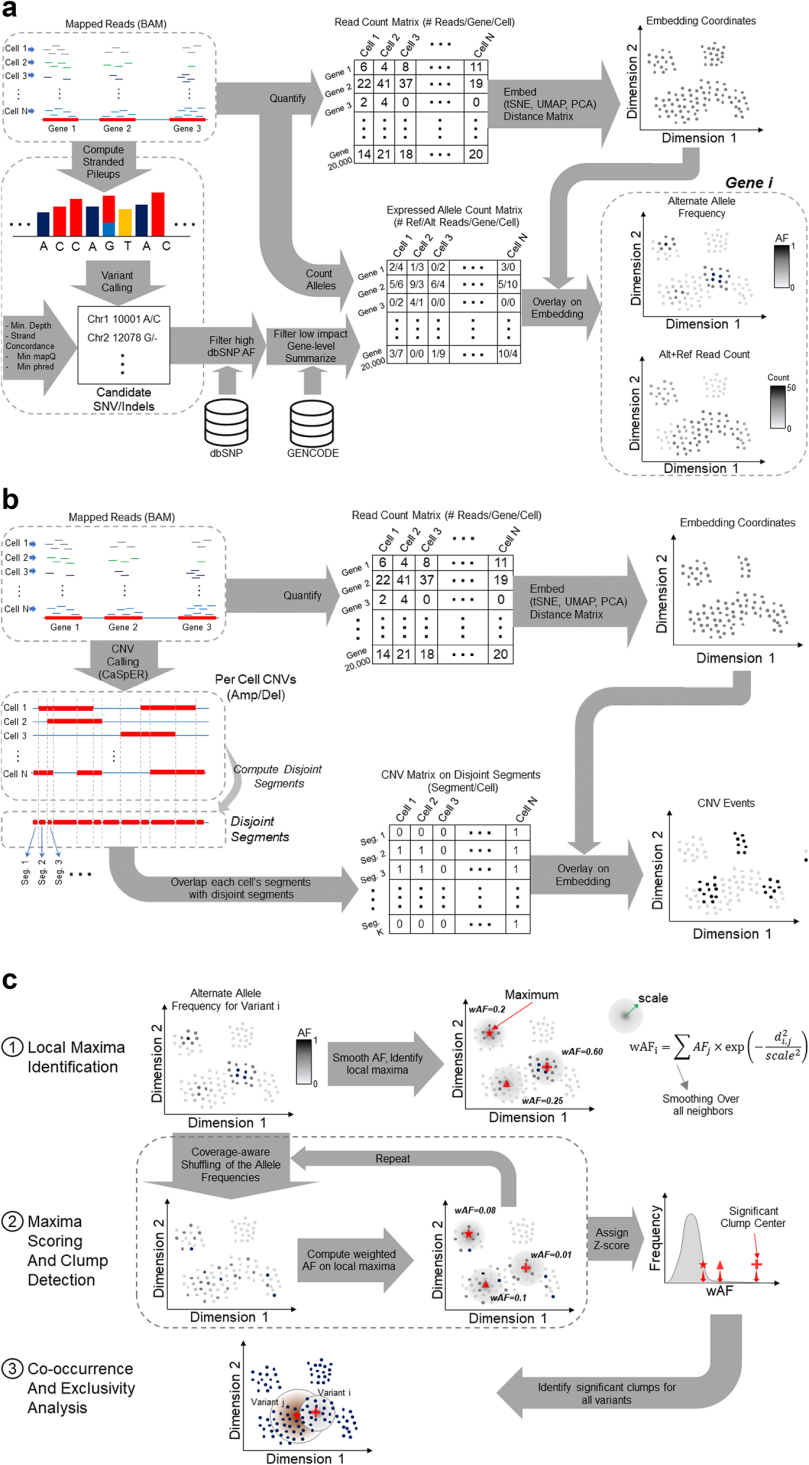
Schematic overview of the XCVATR algorithm workflow. **a** Illustration of single nucleotide variant (SNV)/ insertion and deletion (indel) processing steps. The reads (blue dashes) are used to generate strand-specific pileups (only one strand is shown). These pileups are used to detect variants and generate a list of candidate SNVs and indels. Variants are filtered with respect to the Single Nucleotide Polymorphism Database (dbSNP) and their impact on protein-coding genes. Next, reads supporting reference and alternate alleles are counted for each variant in each cell; counts are stored in the allelic count matrix and are used to estimate variant read-level allele frequencies (AFs). In parallel, the reads mapping to each gene for each cell are counted in the quantification matrix, which is used to compute the cell embeddings and the cell–cell distance matrix. Variant AFs are mapped onto the embedding coordinates for visualization. **b** The illustration of the copy number variation (CNV) processing steps. The CNV segments are pooled, and the breakpoints are pooled and sorted on each chromosome separately. Consecutive breakpoints are used to define the set of minimal CNV segments that do not overlap with any breakpoint. Each new CNV segment is assigned a value of 0 or 1 for each cell based on their amplification/deletion (amp/del) status in that cell; these data are used to generate the binary matrix, which is mapped on the embedding to visualize clumps. **c** Clump detection steps. Read-level AFs are first smoothed on the embedding coordinates, and local maxima representing the clump centers are identified. This is followed by assignment of *z*-scores by read-depth (RD)-aware shuffling. Finally, the significant clumps are compared and visualized

Figure 1 outlines the steps of the XCVATR algorithm, which are summarized below (see Methods for more details). Input to XCVATR is a mapped-read file, such as a BAM file. While this is typical for single-cell (sc)RNA-seq data, bulk datasets contain many BAM files (i.e., one for each replicate), and these can also be used in the analysis without any extra pre-processing.

By default, XCVATR relies on the distance matrix between cells (or samples) that is generated based on transcriptomic profiles. The first step in constructing this distance matrix is read count quantification for each cell (or sample), which is used for computing either the embedding coordinates of the cells or building the distance matrix directly from the expression levels. It is worth noting that XCVATR can also make use of distance matrices that are computed by other means, such as proteomic distances that are estimated from measurement of protein expression levels [30, 31].

Next, XCVATR performs detection and annotation of the genetic variants. Variant detection is designed to include an integrated and flexible single nucleotide variant (SNV)/insertion and deletion (indel) calling step into XCVATR’s variant clump detection analysis. XCVATR generates pileups to identify candidate SNVs, which are then passed through several filters, such as total coverage, strand bias, and mapping qualities. This strategy is similar to that used by the VarScan suite of variant callers [32, 33]. Notably, variant detection can also be parametrized in a relaxed manner, allowing users to evaluate clumps for variants that may be missed with conventional pipelines (e.g., variants with low AFs). We hypothesized that such relaxed variant calling can still be meaningful, as the variants will be further filtered in the context of variant clumping analysis in XCVATR. We therefore suggest that variant calls from XCVATR should only be used for clumping analysis and not for other downstream analyses. However, existing variant call sets (e.g.*,* VCF files) generated by other pipelines, such as GATK [34] and Mutect [35], can be provided as input, thereby skipping the variant detection step.

Variant annotation is integrated into XCVATR to make the workflow more flexible and complete. This is because although there are well-established protocols for variant annotation, such as VEP [36] and AnnoVar [37], these methods occasionally change over time, making it challenging to integrate their output and provide reproducibility. Therefore, XCVATR performs variant annotation to provide a stable and flexible filter for selecting variants with respect to impact. XCVATR takes the variant annotation file (e.g.*,* GTF or GFF) and annotates the detected variants with respect to their impact on the protein sequence. These variants are then filtered to identify and include the most impactful mutations (See Methods for more details on annotation and variant calling filters).

#### Allele counting

For SNVs and small indels, XCVATR counts the number of mapped reads for each variant that support the alternative and reference alleles (Fig. 1a). Using this information, XCAVTR builds a matrix and computes the estimated alternative AFs of each variant. These are then used as scores to assess a variant’s existence in each cell.

For CNVs, XCVATR relies on an existing call set. CNVs are first separated into amplifications and deletions and then analyzed at two different scales. This is because CNVs are distinct compared to small variants, in that they can cover large domains that are as long as chromosomal arms. Therefore, to analyze CNVs at different length scales, XCVATR performs clumping analysis at both large scale (i.e., chromosome arm-length scale) and at segment-level scale. Large-scale CNV analysis includes possible deletion and amplification events for each of the 44 chromosomal arms. For these, XCVATR first builds a binary count matrix that is analogous to the alternative allele count matrix generated for small variants (Fig. 1b). Each entry in this matrix indicates the existence of the CNV (row) in the corresponding cell (column). At the segment-level scale, each CNV is treated as a separate variant. However, because the CNVs identified in each cell can overlap, XCVATR first identifies the smallest set of non-overlapping CNV segments by overlapping the CNVs from cells and identifying the minimal set of common variants (Fig. 1b). Next, these common variants are used to build a binary count matrix similar to the large-scale matrix. XCVATR counts each of the common and disjoint events as a separate variant and performs variant clump analysis.

After the alleles are counted, XCVATR can optionally analyze the variants by identifying and selecting the most impactful variant in each cell (or sample) for each protein-coding gene. This will filter out many variants and provide the user with a clearer view of the clumps at the gene level. In addition, this gene-level summarization of the annotations is important when different mutations in a driver gene can display the same clumping behavior (See Analysis of Bulk Meningioma RNA-seq Samples).

#### Aggregated rare variant summarization and visualization

We hypothesized that variants with low population frequency, by default using The Single Nucleotide Polymorphism Database (dbSNP) [38], are enriched among sample-specific (i.e., *de-novo* or singleton) mutations and somatic mutations (in the case of neoplastic samples). Additionally, sample-specific variants should be present at similar proportions in normal cells relative to tumors or any type of neoplastic cell. To visualize the rare variant fraction in each cell, XCVATR uses the rare variant read-level AF call matrix and computes the fraction of expressed alternative alleles of rare variants among the accessible rare variants. In this way, XCVATR generates a normalized measure of rare variant content in each cell’s expressional state. This aggregated rare variant summarization and visualization step may help to incorporate population-level variant frequency information into the embeddings (i.e.*,* transcriptional states). XCVATR can also visualize existing metadata, such as assigned cell types, jointly with the aggregated rare variant fractions.

#### Smoothing scale selection on the embedding

XCVATR next performs a multiscale analysis of the distance matrices to identify variant clumps. This approach is similar to the multiscale filters that are used to identify blobs in images [39–41]. Each scale defines a neighborhood around a cell in the embedding coordinates and is used to smooth the AFs with a Gaussian filter that is centered on the cell and decreases with increasing distance from this center cell. The scales, however, must be tuned to the distance metric or the embedding coordinates. XCVATR performs a scale selection to tune the analysis to the selected cell–cell distance metric. For each cell, XCVATR identifies *N*_*ν*_ cells that are closest to it (i.e.*,* neighbors), thus defining the close neighborhood of each cell. XCVATR then scans neighborhood size, as follows:

$$\left(0.1\times {N}_{cells}\right)>{N}_{\nu }>\max \left(0.01\times {N}_{cells},10\right)$$

selecting those that include between 1% (or 10 cells, if lower) and 10% of cells in the sample (*N*_*cells*_). It then computes the minimum and maximum radii for each cell that satisfies the above condition. XCVATR identifies the medians of the minimum and maximum radii over all cells and uses these final minimum and maximum radii (*σ*_*min*_, *σ*_*max*_) for multiscale analysis. This computation can be performed efficiently, since the distance matrix (unless it is provided) can be computed quickly from the embedding coordinates using fast matrix multiplications. Neighbor detection is then performed by sorting the distances and selecting the closest *N*_*ν*_ cell (or samples). After this step, only the closest neighbors are processed by XCVATR.

#### Variant clump candidate selection

One of the challenges in clump detection is the large number of cells that need to be analyzed in different scales. To decrease the search space and reduce the cost of modeling, XCVATR performs a cell-centered analysis, wherein it does not aim to model the whole embedding space, but rather focuses on the cells, such that each detected clump is centered around a specific cell (or sample). This is a reasonable expectation, as the expected clumps are larger than the cell–cell distances, and therefore, clump detection should be accurate even when clumps are centered around cells.

From visual evaluation of the variant AF distributions on embeddings, the number of clumps were observed to be much smaller than the number of samples or cells. Motivated by this, we designed a clump-center-candidate pre-selection that decreases the search space for the clump centers. Given a smoothing scale *σ*_*a*_ at the scale *a*, XCVATR computes a smoothed AF value for each cell, as follows:

$${\overline{\phi}}_i^{(a)}=\frac{1}{\left|{N}_{\nu}^{(a)}(i)\right|}\times {\sum}_{k\in {N}_{\nu}^{(a)}(i)\backslash i}{\phi}_j\times \exp \left(-\frac{d_{i,j}^2}{\sigma_a^2}\right)$$

where *ϕ*_*j*_ denotes the alternative AF of the variant in the *j*^*th*^ cell (1 > *ϕ*_*j*_ > 0), *d*_*i*, *j*_ denotes the distance between the *i*^*th*^ and *j*^*th*^ cells in the sample, and ${N}_{\nu}^{(a)}(i)$ indicates the set of indices for the cells that are in the vicinity of the *i*^*th*^ cell for the scale *a*. From the above equation, the smoothed AF of the *i*^*th*^ cell, ${\overline{\phi}}_i^{(a)}$, is higher when its neighborhood contains many cells with high AFs. In addition, the smoothed AF depends on the scaling parameter *σ*_*a*_, and each scale is processed independently from other scales. XCVATR then identifies potential variant clump centers in the embedding coordinates by determining the set of cells whose neighborhoods contain cells that are strictly lower in terms of smoothed AF (Fig. 1c), that is, the cells whose smoothed AF is the “local maximum”. These cells are considered candidates:

$${C}_a=\left\{i\left|\forall j\in {N}_{\nu}^{(a)}(i):{\overline{\phi}}_i^{(a)}>{\overline{\phi}}_j^{(a)}\right.\right\}$$

where *C*_*a*_ denotes the indices of cells that are clump centers. An advantage of this multiscale processing is that the above condition does not rely on any thresholding or modeling and fits naturally to the distribution of smoothed AF data on the embedding. In addition, we observed that the number of locally maximal clump center cells is at least 1–2 orders of magnitude smaller than the number of cells. Thus, the number of clumps that need to be scored decreases substantially with little impact on accuracy (See below).

#### Specification of position and size of clumps

Up to this point, we have identified each clump by the cell at its center, which specifies the position of the clump in the embedding space. In addition to the center, it is also necessary to define the radius of the clump, so that its size can be determined. For this, XCVATR makes use of the scale parameter at which the clump is identified (i.e.*, σ*_*a*_ at scale *a*). Thus, all cells that are closer than *σ*_*a*_ to the center of a given clump are assigned to this clump.

#### Variant clump evaluation by read-depth (RD)-aware permutation

For each candidate clump center cell in *C*_*a*_ (*a*^*th*^ scale), the corresponding smoothed AF is compared to an empirical background. To this end, XCVATR utilizes a permutation test to assign significance to each candidate clump center by permuting the AF of all cells (including non-candidates) and computing the smoothed AF (described above) for each candidate with permuted AF’s. This is repeated *Κ* times. For each permutation, the smoothed AFs are computed for every candidate clump center, and this is used to build an empirical background (Fig. 1c). XCVATR then computes a *z*-score that is used to rank the clumps and assigns final scores, as follows:

$${Z}_{\phi }=\frac{{\overline{\phi}}_i^{(a)}-{\mu}_{\phi }}{\delta_{\phi }}$$

It is important to ensure that the AF provides new information and does not simply recapitulate the geometry imposed by the embedding coordinates. This may occur, for example, when some cells are expressing a cell-specific marker that is not expressed at all in other cells. In this case, a non-impactful germline variant may be expressed in this cell population, whereas other cells will show no expression of the variant. In such a scenario, a naïve approach would determine that this variant exhibits a clump comprised of the cells where the gene is expressed. This would be an uninteresting clump that emerges based on the cell-type specificity of the gene. To filter out these clumps, XCVATR sets a threshold, *τ*, on the total read depth at which a variant (Sum of reference and alternate read counts) is expressed in each cell and estimates a *z*-score using this read depth as a co-variate. This allows the clumps to be evaluated and filtered with respect to RD bias. In order to further filter the clumps, XCVATR also computes the significance of enrichment for expressed alternative alleles at both the read level and the cell (or sample) level in each clump.

#### Assessment of read-level alternate allele expression enrichment in clumps

Given the clumps identified for a variant, XCVATR first computes the total number of alternate and reference reads from all cells (i.e., counts in the bulk sample). These bulk-allele counts are used as a baseline alternate AF for the corresponding variant, which we denote by $A{F}_{alt}^{(bulk)}$. Next, for each clump, the total alternative allele supporting reads and total reads are computed using only the cells in this clump. At scale *a*, for the *b*^*th*^ clump, the allelic counts are used to compute the read-level modified binomial *P*-value, with bulk alternate allele frequency is used as the flipping probability, using the binomial function $Bin\left({n}_{alt}^{\left(a;b\right)},{n}_{ref}^{\left(a;b\right)};p=A{F}_{alt}^{(bulk)}\right)$, where ${n}_{ref}^{\left(a;b\right)}$ and ${n}_{alt}^{\left(a;b\right)}$ denote the number of reads supporting the reference and alternate alleles, respectively, for the corresponding variant. This binomial *P*-value estimates the significance for enrichment of alternate allele-supporting reads in clump *b*, when compared to randomly assigning reads to all cells with the probability, *p* = *AF*_*bulk*_.

#### Assessment of cellular-level alternate allele expression enrichment in clumps

Next, XCVATR computes enrichment of alternate AFs at the cell level. At scale *a*, XCVATR counts the cells in clump *b* having alternate AFs above *η*. XCVATR then counts the number of cells in the whole sample for which the alternate AF is above *η*. These values are used to compute significance for enrichment of alternate alleles at the cell level with Fisher’s exact test, by building a contingency table of cell counts in the bulk sample and in each clump, as follows:

**Table:** 

Variant ***k***	Number of cells whose AF > ***η***	Number of cells whose AF < ***η***
**Clump** ***b*** **identified at scale** ***a***	${c}_{\eta}^{\left(a;b\right)}(j)$	${c}_0^{\left(a;b\right)}(j)-{c}_{\eta}^{\left(a;b\right)}(j)$
**Bulk**	${c}_{\eta}^{(bulk)}$	${c}_0^{(bulk)}-{c}_{\eta}^{(bulk)}$

where ${c}_{\eta}^{\left(a;b\right)}(j)$ indicates the number of cells in clump *b* at scale *a*, with AF > *η*, and ${c}_{\eta}^{(bulk)}$ indicates the number of all cells (i.e., bulk sample) for which AF > *η*. Fisher’s exact test evaluates enrichment of a clump’s AF statistics in comparison to the whole bulk sample. In particular, this test filters out cases where one cell in a small clump displays a high AF, while other cells in the clump express the alternate allele only at low levels, which may still pass the read-level enrichment filter. Thus, the read-level and cell-level enrichment estimates are used to filter out clumps (with *P* < 0.05) exhibiting low levels of enrichment in comparison to the bulk sample at the read and cell (or sample) level.

Finally, XCVATR computes the effective radius for each clump by iterating over the cells closest to the clump’s center cell. In this way, cells in the neighborhood surrounding the clump’s center are analyzed as they expand in radius. For each neighborhood, the area where cell-level enrichment is maximized (i.e., Fisher’s exact test *P*-value is minimized) is selected as the effective radius of the clump. Finally, the clump centers and the scale at which they are identified, permutation *z*-scores, alternate allele enrichment statistics, and effective radii are reported in the output.

#### Visualization

XCVATR provides visualization of the clumps on the embedding coordinates for each variant. This enables users to manually evaluate the variants and can also be helpful for visualizing cell-type specifications and phenotypic properties in relation to the clumps. Visualization utilities are implemented in R and directly make use of the data generated by XCVATR.

### Analysis of detected variants and clump testing

**Fig. 2:**
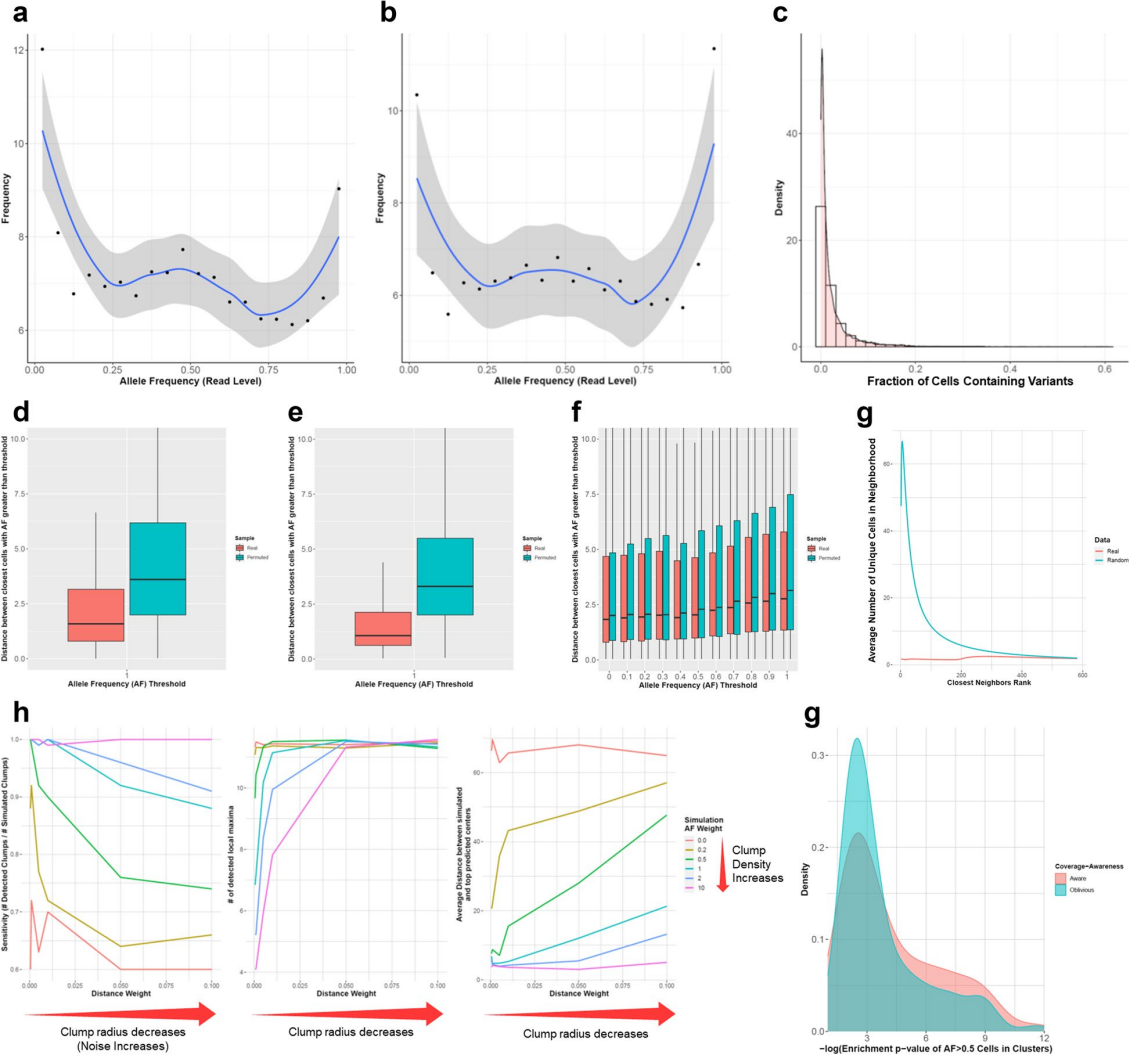
Clumping and variant statistics for existing RNA-sequencing (RNA-seq) datasets. **a** Read-level alternative AF distribution of variants from bulk meningioma RNA-seq data [42]. **b** Read-level frequencies of variants from single-cell (sc)RNA-seq data using the BT_S2 sample glioblastoma sample from Darmanis et al. [43] **c** Distribution of the fractions of cells containing variants in single-cell data; the x-axis shows the fraction of cells that contain variants, and the y-axis shows the density. **d** Clumping statistics for large-scale deletions and for **e** large-scale amplifications. Boxplots show the distribution of distances between the nearest CNV-harboring cells (red) and same plot with shuffled AF data (blue). **f** Clumping statistics for SNVs/indels in the BT_S2 dataset; x-axis shows the read-level AFs for selecting cells used in the analysis. Boxplots are shown for each AF cutoff, corresponding to read-level (red) and AF-shuffled (blue) data. **g** Number of unique cells (y-axis) at closest-neighbor rank in neighborhoods of increasing size (x-axis) for the BT_S2 dataset over 100 independent t-distributed stochastic neighbor embedding (t-SNE) runs generated by SEURAT. Numbers of unique neighbors for randomized (blue) and real data (red) are shown. **h** Simulated clump detection accuracy. Clump detection sensitivity (left), number of detected clumps (middle), and distance between real and detected clumps are shown (right); x-axes show the distance weight, and colors indicate the AF weight used for clump simulation. **i** Distribution of variant-harboring cell enrichment, with (red) and without (blue) RD-aware shuffling for clump detection

To investigate the frequencies of identified variants from existing RNA-seq data, we measured the alternative AFs of detected variants in bulk (from 160 meningioma patients [42]) and scRNA-seq datasets (BT_S2 glioblastoma sample from Darmanis et al. [43]) using XCVATR. We generally observed that the detected variants in the bulk dataset exhibit an AF spectrum that is dominated by alternate AFs of 0 and 100% (Fig. 2a), with a slight enrichment at 50%. For the single-cell dataset, consistent with previous studies [26], we found that a substantial proportion of the mutations are expressed in a small fraction of cells (Fig. 2b,c). Thus, our results provide evidence justifying the development of XCVATR.

We next tested our hypothesis that variant clumps are present in t-SNE embeddings. Although we can manually observe these clumps, it is useful to have automated and objective tools for validating their existence and measuring their prevalence. To determine whether such variant clumps frequently occur in embedding coordinates, we computed the average distance between cells containing variants with high alternate AFs. If these cells are closer to each other than expected by random chance, this provides empirical evidence for detectable general clumping behavior of variants. Using the Darmanis et al. BT_S2 dataset [43], we computed the distribution of distances between the closest cells that contain variants with alternate AFs greater than *η*. We also computed the same distribution in shuffled data, such that the AF shuffling is performed in a RD aware manner to prevent RD bias (Fig. 2d–f). We found that for SNVs/indels, as the AF cutoff, *η*, increases, cells containing variants with high alternate AFs are closer to each other relative to the shuffled data (Fig. 2f). Notably, this clumping behavior is much clearer when we performed the same analysis with CNVs (Fig. 2d,e). In this case, we computed the cell–cell distance distributions for cells containing CNVs (i.e., amplifications and deletions) and found that the distribution of distances is much smaller in the real compared to the shuffled datasets for both deletions and amplifications (Fig. 2d,e). This observation that CNVs exhibit a much stronger clumping effect than SNVs and indels may result from the fact that CNVs have a stronger impact on the transcriptional state of the cells. Critically, these results provide evidence for the general clumping of cells with respect to SNV, indel, and CNV frequencies.

### Robustness of local statistics

We further evaluated the robustness of local distance statistics from the t-SNE coordinates that are used for embedding of scRNA-seq data. This is necessary because as a probabilistic embedding technique, t-SNE requires a seed for the pseudorandom-number generator [44]. When the seed is changed, or when t-SNE is ran twice with the same seed, the embedding coordinates are changed. However, robustness of sample locality is essential for XCVATR clumping analysis, as this algorithm is designed to detect the local clumping of cells harboring variants with high alternate AFs. Therefore, to test the local robustness of t-SNE embedding coordinates, we first ran t-SNE on the Darmanis et al. dataset [43], using the SEURAT package [45] with default parameters. The t-SNE coordinates are generated 100 times, changing the seed number with every run. Next, for each cell, we identified neighborhoods of increasing size, containing up to 600 of the closest neighboring cells (out of 1170 cells total). For each of the 100 embeddings, we computed the number of unique cells within these neighborhoods and plotted the average number of unique cells at each neighborhood size (Fig. 2g). We also compared this locality information with an identifier-shuffled dataset, where we permutated the cell identities at each embedding and then computed locality preservation for each neighborhood size. We found that, compared to the shuffled data, real data show strong preservation of the embedding. At each neighborhood size, which determines the rank or strength of locality, the real dataset contains, on average, between one and three unique cells among their closest neighbors. In addition, this average does not change with increasing neighborhood size (i.e.*,* increasing closest-neighbor rank in Fig. 2g). For shuffled data, the average number of unique cells is as high as 60 cells at small localities. It should be noted that these results only attest to the robustness of locality statistics. For neighborhoods covering more than 25% of the cells, the randomized and real locality statistics are very similar, which suggests that clumps containing more than 25% of the cells (i.e.*,* neighborhoods with approximately 300 cells in Fig. 2g) may be non-robust and non-reproducible between different runs of t-SNE.

### Accuracy of clump detection

Next, we tested the accuracy of our multiscale clump detection approach by simulating clumps and running XCVATR to detect these simulated clumps. To ensure realistic data, we simulated variant clumps on the t-SNE embedding coordinates with the AFs of known variants identified within the 1170 cells from Darmanis et al. [43] (See Methods). For this analysis, a cell is randomly selected and designated as the known clump center. We use two parameters to define a clump: the first is the scale parameter, which determines the radius of the simulated clump, and the second is the strength of the clump (referred to as “AF weight”). AF weight is tuned by a parameter that forces high AFs to be assigned closer to the center of the simulated clump and thus determines strength of the AF distribution around the clump. Given the clump center cell, we assign a “sampling weight” to every other cell, which depends on the distance to the center cell and the AF of the current cell. These sampling weights are used to shuffle the AFs on the embedding, such that cells with high sampling weights (i.e., close to the clump center and having a high AF) are shuffled close to the clump center (See Methods). This simulation strategy allows us to make use of existing data rather than generating synthetic datasets, which can introduce synthetic biases.

We simulated five different distance weights and six different AF weight parameters, and for each parameter combination, we chose 100 randomly selected clump centers. To illustrate the effect of the parameters, consider two cells whose respective AFs are 0.99 and 0.01. The simulation assigns sampling weights equal to 0.997 and 0.238, respectively, when (AF weight, distance weight) is set to (0.4, 0.005) and distance to the clump center is at 5% of the embedding radius. When the distance weight is increased to 0.05, the sampling weights are 0.999 and 0.858, respectively, indicating that the cells have a more similar probability of being sampled at this distance. When AF weight is decreased to 0.2, the sampling weights become almost identical at 0.999, and 0.926, respectively. Thus, shuffling generates non-trivial and challenging clumps for testing XCVATR.

For each simulation, an alternate read count matrix is generated from the shuffled AF data for the 1170 cells in the simulated dataset and is input to XCVATR. Accuracy of the simulation is then evaluated by comparison of the known clump centers to the clump centers detected by XCVATR. Any clump that is detected within less than 1% of the whole embedding space radius is deemed a match. We evaluated the fraction of times XCVATR was able to correctly identify the cells at the center of the clumps (Fig. 2h, left) and recorded the number of clumps identified by XCVATR (Fig. 2h, right). In addition, we measured the distance between simulated and detected clumps (Fig. 2h, right). Overall, we found that XCVATR can accurately identify and summarize clump centers, with a sensitivity higher than 90% for most parameter combinations, and the number of clumps is bound at fewer than 15 clumps. For less challenging cases (i.e.*,* low distance weights and higher AF weights), we observed an increase in detection accuracy, fewer total detected clumps, and decreased distance between simulated and predicted clumps.

We also evaluated how RD-aware permutation impacts the identified clumps. To this end, we identified clumps from the Darmanis et al. dataset [43] with and without RD-aware shuffling. We then plotted the distribution of cell-level enrichment of alternative alleles, as determined by Fisher’s exact test *P*-values, for the clumps detected with and without RD-aware shuffling (Fig. 2i). We found that RD-aware shuffling enables detection of clumps with a greater enrichment for cells expressing alternative alleles. This analysis provides evidence that RD-aware shuffling can be helpful for identifying clumps that are enriched for cells expressing alternative alleles.

### XCVATR analysis of a glioblastoma scRNA-seq dataset

To demonstrate data analysis with XCVATR, we first used the dataset generated by Darmanis et al. [43], which contains scRNA-seq of tissue from four patients with glioblastoma brain tumors, sequenced using Smart-Seq2 technology [46]. A key advantage of Smart-Seq2 is that it provides more uniform coverage compared to technologies such as Drop-Seq and 10X Genomics, in which there is a 3′-bias on the transcript [47]. While this bias can potentially affect the variant detection step, XCVATR does not require the variants to be complete, as each one is analyzed independent of other variants.

**Fig. 3:**
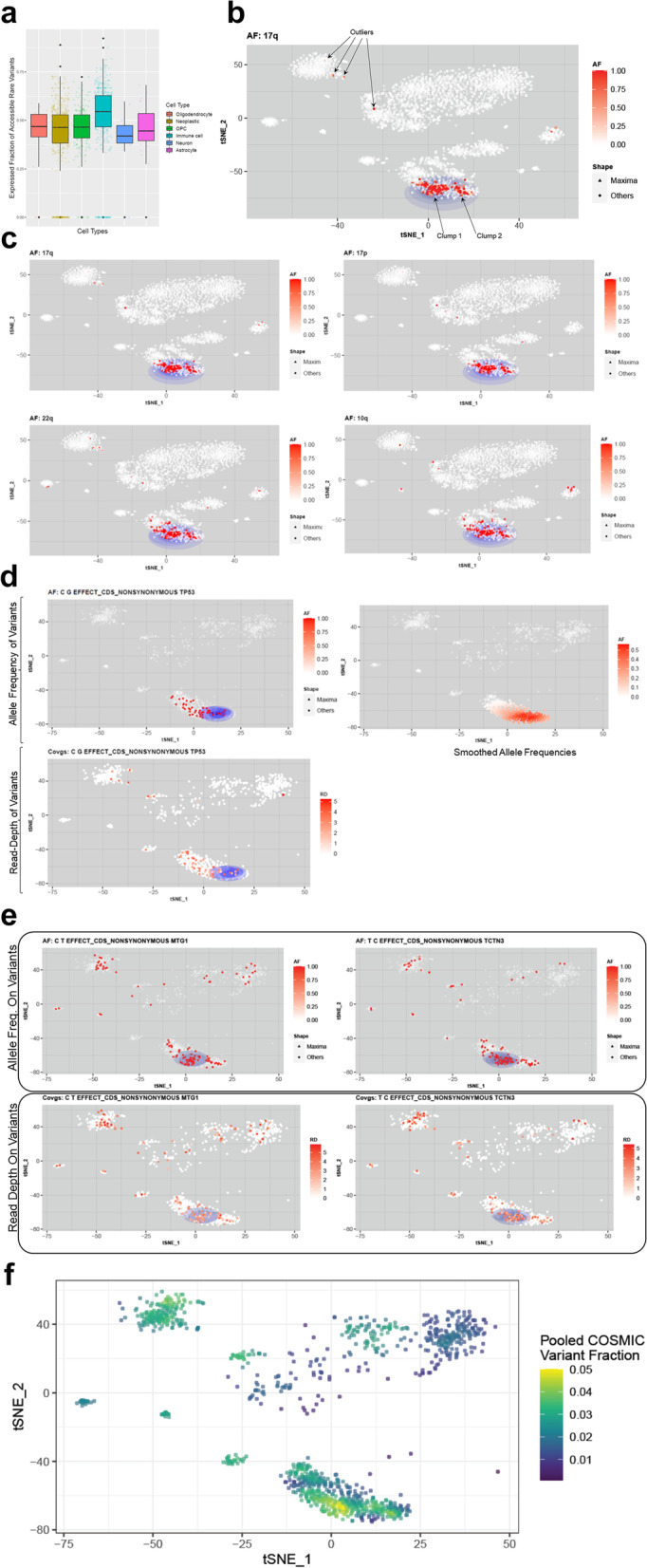
XCVATR analysis of the BT_S2 glioblastoma sample from the Darmanis et al. scRNA-seq dataset [43]. **a** Boxplots showing the distribution of expressed rare allele fractions in the corresponding cell types. Middle whiskers show the median, and the bottom and top whiskers indicate the 1st and 3rd quartiles, respectively. **b** Example of a clump with the 17q deletion. Clumps detected by XCVATR are shown with blue ellipses; each dot represents a cell, with the AF indicated by color (red, high; white, low). **c** Examples of other large deletions detected by clumps; plots show AFs. **d** Mutation in *TP53* (tumor protein 53) that is identified as a clump in the tumor cells. The smoothed AF is shown on the right, and the RD for each cell is shown below. **e** SNVs in the *TCTN3* (tectonic family member 3) and *MTG1* (mitochondrial GTPase 1) genes. Top plots show the AFs, and bottom plots show the RD for each cell. **f** Distribution of expressed COSMIC variant fraction on cells

Here, we found 3590 cells from the metadata (in all samples) that were processed and mapped using Hisat2 and used the embedding coordinates from the t-SNE analysis performed in the original study. After initial inspection of the samples, we focused on one with the id “BT_S2”, which contains the most impactful events in terms of CNVs, SNVs, and indels. After calling variants with XCVATR for 1170 cells, XCVATR identified 201,600 mutations in total that are annotated. After filtering with respect to allele frequencies, 74,575 mutations remained. Further filtering of the mutations with respect to impact, 776 mutations are found to be damaging. The final gene-level summarization of the mutations yielded 687 genes that harbor a damaging mutation (Methods). We first focused on the rare variant fractions in each cell by filtering out variants whose dbSNP population AFs are greater than 1%, as we hypothesized that variants with very low population frequencies are either sample-specific mutations (*de-novo* or singletons) or somatic mutations. We found that rare variants are more highly represented in the immune cells and less represented in the neurons (Fig. 3a).

We next ran XCVATR on the CNVs (identified by CaSpER) and short variants. We focused on the large-scale CNVs and evaluated the initial clump centers detected by XCVATR in the first pass of the multiscale decomposition for amplifications (Fig. S1) and deletions (Fig. S2). As expected, XCVATR decreases the search space substantially by focusing the clump search on a sparse set of cells. After clump scoring, we assessed the final set of clump calls. Figure 3b shows an example of a clump that is detected by XCVATR for a deletion of chromosome arm 17q, which is identified as the top clump among other large-scale deletions. This is a well-known deletion that is observed in glioblastoma tumors, and two clumps with the 17q deletion are identified for this population of malignant cells. We included distributions of representative clumps that are detected for deletions on chromosome arms 17p, 10q, and 22q (Fig. 3c), which are reported as the top CNVs. In addition, XCVATR identified clumps identified by deletions of chromosome arms of 4 and 13 (Table S1) and amplifications of chromosomal arms of 7, 20, and 21 (Table S2), which are frequently altered in tumors. While these results are partially expected, they also corroborate the clump detection performed by XCVATR.

We next analyzed the SNVs and indels identified by XCVATR. XCVATR identified numerous clumps that form in the embeddings at different scales (Table S3). One of the top deleterious variants detected by XCVATR is in *TP53* (tumor protein 53), which encodes the well-known DNA-repair protein p53 (Fig. 3d). This mutation is also marked as deleterious in the Catalogue of Somatic Mutations in Cancer (COSMIC) database [48]. Smoothed AF signals indicate that there is a clear enrichment of the alternate allele in malignant cells. The RD at which each cell harbors the variant is shown in the bottom panel (Fig. 3d), thereby allowing users to manually inspect for RD biases. We also identified variants in several other genes, including *TCTN3* (tectonic family member 3) [49] and *MTG1* (mitochondrial GTPase 1), which form significant clumps on the same set of cells (Fig. 3e). Interestingly, these genes are also mutated in some of the normal cells, as observed on the t-SNE embedding, which could result from misclassification of cells. Critically, these results suggest that clump detection can provide additional insight in analysis of tumor scRNA-seq datasets.

We next focused joint analysis and visualization of multiple variants. This is advantageous to evaluate the effect of a set of variants that can collectively impact global expression levels concordantly and help identify cell types or transcriptional programs. We focused on the COSMIC catalogue of variants as they are most relevant to cancer literature. We calculated the average fraction of the 363 expressed COSMIC variants in each cell and visually evaluated the smoothed fractions on the embedding using XCVATR’s smoothing function (Fig. 3f). We found that there is a clear enrichment of the COSMIC variant expressions that coincide with neoplastic cell populations in the embedding. As a baseline, we selected 363 random variants from the detected variants and performed pooling and computed the expressed fraction of random variants. We repeated this permutation 20 times. Overall, the maximum of the expressed variant fraction is on average much smaller for the randomly selected variants compared to the actual COSMIC variants (0.05 vs 0.0082+/− 0.0012). This result demonstrates that COSMIC variant pooling is substantially different from a permuted set and potentially contains biologically relevant signal. In summary, this analysis demonstrates that analysis and visualization of different variant sets can provide useful insight into detection of different cell types with respect to their expressed variant profiles.

### Copy number variant analysis of a meningioma scRNA-seq dataset

**Fig. 4:**
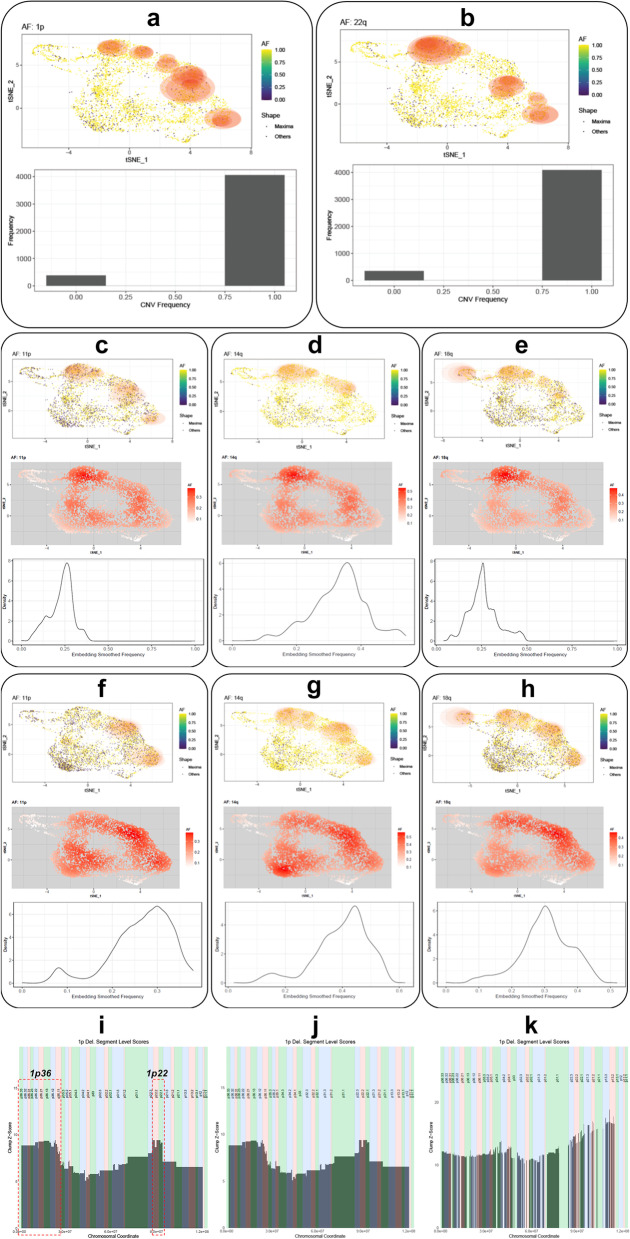
Analysis of the expressed variants and CNV clumps in our meningioma scRNA-seq dataset. Clumps identified for the **a** 1p and **b** 22q deletions. Bottom plots show the counts of cells harboring the CNVs. Clumps identified for the **c** 11p, **d** 14q, and **e** 18q deletions in the frontal sample. Clumps are highlighted by the blue ellipses, smoothed AFs are shown in the middle row, and distribution of smoothed AFs are shown in bottom row. Clumps identified for the **f** 11p, **g** 14q, and **h** 18q deletions in the postal sample. Clumps are highlighted by the blue ellipses, and smoothed AFs are shown in the bottom row. **i** Segment-level deletions on chromosome 1p in the frontal sample; x-axis shows the genomic coordinates, and y-axis shows the *z*-scores assigned by XCVATR to each segment. Cytobands on the genomic coordinates are indicated by rectangles of alternating colors, with the cytoband name shown at top. The segments on 1p36 and 1p22 with higher clumping scores are boxed in red, dashed rectangles. Clumping scores for segments on chromosome 1p in the frontal **j** and postal **k** sample are shown

We next analyzed new scRNA-seq datasets generated by our group that include two samples (referred to as frontal and postal) obtained from the brain of a patient with meningioma tumors. The frontal sample is from the primary meningioma tumor. This primary tumor metastasized within the patient’s brain to another location, and the metastasized tumor was extracted to yield the postal sample. Both samples were sequenced using the 10X Genomics scRNA-seq platform, and reads were mapped with the CellRanger software suite. The SEURAT package was then used to filter out low-quality cells (i.e.*,* those with low coverage, high mitochondrial reads, and potential doublets) and to generate the t-SNE embedding coordinates from CellRanger read counts. We identified CNVs in these samples with CaSpER, and using the embedding data and the CNVs, we performed clump detection in XCVATR. For these samples, we also identified the CNVs from an existing genotyping array of the bulk tumor samples using the DNAcopy algorithm [50]. We analyzed the CNVs identified in both the scRNA-seq and genotyping array datasets and found that tumor cells predominantly contain the 1p and 22q large-scale (i.e., chromosome arm) deletions (Fig. 4a, b). As such, XCVATR does not assign high clumping scores to these deletions.

We then focused on the 11p, 14q, and 18q deletions (Fig. 4c–h) that were identified in both the genotyping array and the scRNA-seq data. XCVATR identified strong overlapping clumps for these deletions, and the smoothed AFs indicate a clear enrichment of these deletions in tumor cells. When clumping patterns in the frontal and postal samples are compared, notable differences are observed, with the postal samples (Fig. 4f–h) showing additional clumps compared to the frontal samples (Fig. 4c–e). These clumping differences in frontal and postal samples are more visible when the smoothed AF plots are compared (Fig. 4c–h). Based on these observations, we hypothesize that differential or comparative visualization analysis with XCVATR can provide a complement to existing computational methods and yield additional insights.

We also examined segment-level events in the frontal and postal scRNA-seq samples using XCVATR. For this analysis, we used the segment-level CNVs reported by CaSpER and visualized these events in XCVATR by mapping the clumping *z*-scores on the genomic coordinates. Among the 5623 deletion segments, in the frontal sample, we found that segment-level deletions at 1p.36 (a well-known locus deleted in tumors) exhibit higher clumping scores relative to the other segments in chromosomal arm 1p (Fig. 4i). Additionally, deletions in a relatively short segment covering 1p.22.1 and 1p.22.2 (described previously) are assigned high clumping scores in the frontal sample (Fig. 4j). However, these regions do not exhibit high clumping scores in the postal sample (Fig. 4k). Chromosome arm 22q exhibits more uniform clumping behavior [51] in both the frontal and postal samples, whereas arms 14q and 18q show differences in clump scores at the segment level.

We also analyzed the potential impact of dropout events on clump identification. Drop-outs refer to observation of zero read count on genes and are caused by stochastic sampling of RNA molecules from the library. While drop-outs have been treated wide as technical factors, number of studies have found that they provide biologically useful information about cell clustering [52] (Additional file 1), and identification of rare cell types. We estimated drop-out rates for each chromosome in our samples by calculating the fraction of protein-coding genes with exactly zero RNA-seq reads in each cell and found approximately 86% overall drop-out rate. When dropouts are estimated for each chromosome, we found that dropout events reflect the deletion patterns on the chromosomes 22, 10, 18, 14, 11, and 1 (Fig. S3).

### XCVATR analysis of bulk meningioma RNA-Seq samples

**Fig. 5:**
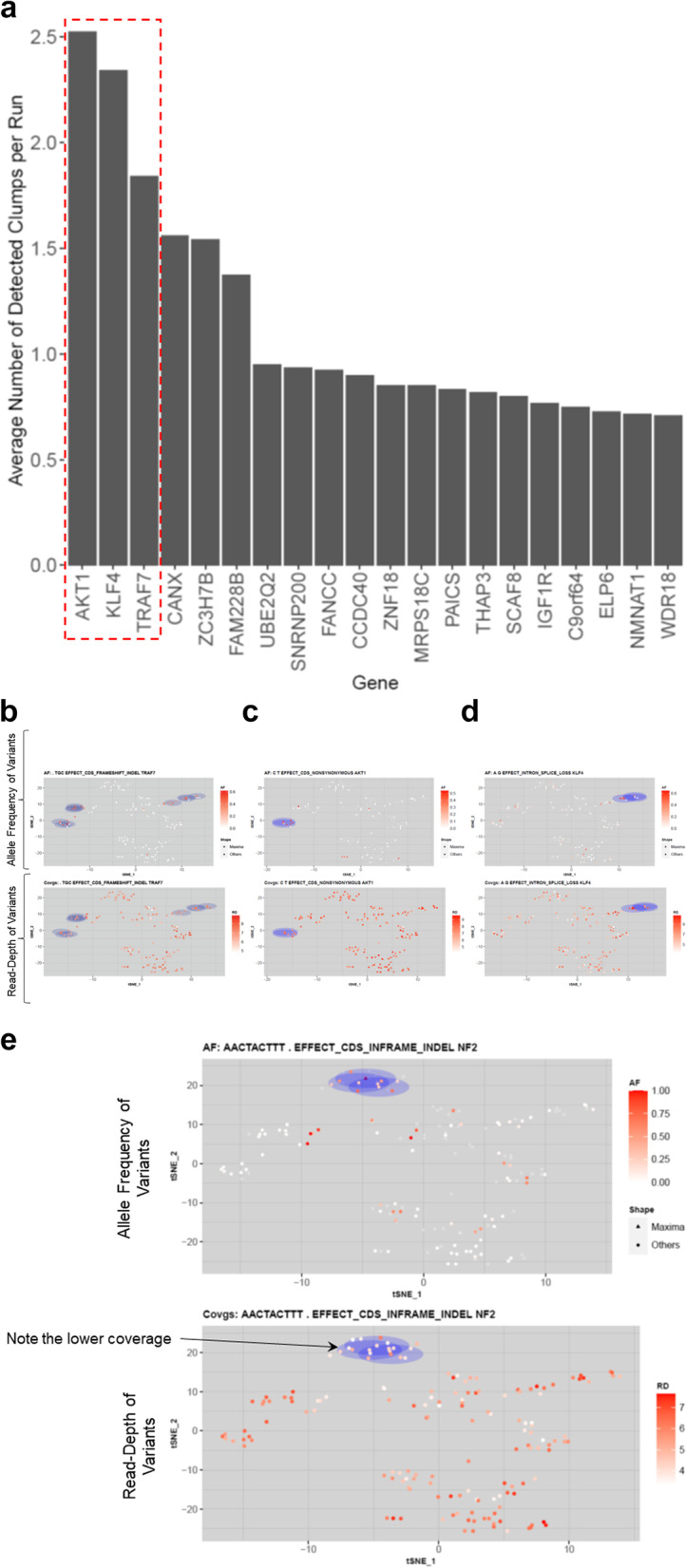
Analysis of an existing bulk RNA-seq dataset containing 160 meningioma samples. **a** Graph showing the average number of clumps for each gene detected by XCVATR with 180 different t-SNE parameter combinations. Genes are shown on the x-axis, and the average hits for each gene are shown on the y-axis. The top-three genes are boxed in the dashed, red rectangle. Clumps detected by XCVATR for the **b** *AKT1* (AKT serine/threonine kinase 1), **c** *TRAF7* (TNF receptor-associated factor 7), and **d** *KLF4* (Kruppel-like factor 4) genes. Read coverage is shown at bottom. **e** Clumps associated with mutations in the *NF2* gene. Read coverage is shown in the bottom plot

Lastly, we used XCVATR to analyze SNVs and indels in an existing bulk RNA-seq dataset obtained from a cohort of 160 meningioma patients [42]. Variants were detected and annotated with XCVATR (See Methods) and filtered with respect to impact and population frequency (See Methods); identified variants were summarized to gene-level events. We then constructed the gene expression matrix, performed t-SNE to generate the embedding coordinates of the data, and ran XCVATR to identify strong variant clumps. In order to evaluate the effect of t-SNE parameters (i.e.*,* the perplexity parameter, number of top variable genes, and minimum expression cutoff) on clump detection, we ran t-SNE with 180 different parameter combinations and then ran XCVATR with each of the resulting embeddings. Analysis of the genes associated with the top-scoring clumps revealed that these clumps are associated with mutations in the *KLF4* (Kruppel-like factor 4), *AKT1* (AKT serine/threonine kinase 1), and *TRAF7* (TNF receptor-associated factor 7) genes (Fig. 5a–d). These were the genes most frequently reported by XCVATR among the 180 different embedding parameter combinations used, and notably, mutations in these genes are extensively reported as recurrent events in meningioma tumors [53]. In contrast, clumps associated with mutations in *NF2* (neurofibromin 2), a common driver of meningioma, scored lower in the XCVATR clump analysis (Fig. 5e). Upon inspection of the data, we conclude that this likely results from low coverage of the *NF2* gene in most samples, which also contain chromosome 22 deletions. Overall, these results provide evidence that XCVATR can help to uncover biologically relevant mutations in bulk RNA-seq samples.

## Discussion

In this study, we present XCVATR, a method that analyzes the spatial enrichment of expressed variant alleles in bulk and scRNA-seq data to identify local clumps in cell and sample embeddings. XCVATR makes use of local spatial geometry from the embedding and multiscale analysis to provide a comprehensive workflow for detecting expressed variant clumps. By visualizing these clumps on the embeddings, they can provide insight into driver genes and key mutations underlying pathophysiological states.

Critically, to provide maximal levels of user control, XCVATR integrates variant detection, annotation, filtering, and clump detection in one package and therefore does not explicitly require any particular variant-calling methods, such as GATK [54]. We believe that this high level of control on variant calls is especially important to make variant calling as relaxed as possible and provide a comprehensive set of variant calls, which can then be stringently filtered at the clump-detection stage. This allows rare somatic variants in cancer samples to be more inclusively analyzed relative to existing pipelines that may miss rare variants with their default calibrated parameters [55].

One of the current limitations of our method is that the concept of a clump needs more refinement and should be more discretely defined (Additional file 1). In addition, the different embedding strategies should be surveyed to evaluate how embedding strategy and dimensions impact clump identification. The concept of a clump is similar to peak in ChIP-Seq analysis (1D clumps) [56, 57] and blob detection (2D clumps) in image analysis [41, 58]. New methods can be proposed that make use of multiscale decomposition of graphs using Gaussian and wavelet-based techniques to build multiscale pyramid representations and statistical modeling of the clumps in these representations. On a separate note, the technical factors such as drop-out can have complex impact on detection and analysis of CNVs and should be carefully analyzed and interpreted because they can provide useful biological insight into detection of malignant cells and rare cell populations [52].

It is useful to place XCVATR in the context of other tools that use variants detected from RNA-seq data for downstream applications, such as building phylogenetic trees and cell clustering [27], identifying phenotype-genotype associations [59], and measuring allele-specific expression [60]. Critically, although variant detection and variant-level analysis of scRNA-seq data have been reported in a number of studies, XCVATR can be distinguished from other available methods based on two major differences. First, these methods require variants to be identified a priori using specialized pipelines that require variant callers, such as GATK or Mutect. However, we believe that these pipelines can be too stringently tuned, and thus may miss low-frequency variants, such as those present in tumor samples [61]. This is reasonable since these pipelines aim to identify variants that can be used in any context. Appropriate stringency is particularly important for detecting somatic variants vs. germline variants (i.e.*,* those present in normal tissues), as somatic variants may be expressed at very low AFs in RNA-seq data. To circumvent this, XCVATR utilizes a general and relaxed variant detection approach and allows user control of the SNV/indel detection parameters. In addition, these variants are used only in the context of clump detection and are filtered by the XCVATR downstream clump-detection criteria. We therefore expect that XCVATR will be most useful for samples with high somatic heterogeneity, such as tumor samples.

A second feature that distinguishes XCVATR from other methods is that many of these existing approaches focus primarily on SNVs and indels, with only very limited attention to CNVs. Although these other methods could be adapted to cluster cells with respect to CNVs, this is not a trivial change, as CNVs exhibit very different statistics compared to SNVs/indels. We have accounted for these differences in XCVATR and included CNV analysis as an integral part of our pipeline. Overall, we show that XCVATR provides a complete workflow for detection, annotation, filtering, analysis, and visualization of large- and small-scale variants in both bulk and scRNA-seq data, which is advantageous for ease of usage and installation. Further, our data suggest that XCVATR can provide valuable insight into associations between variant alleles and cellular transcriptional states, particularly for heterogeneous samples containing rare variants, such as tumors.

## Conclusions

Detection of variant association within expression embedding provides important insight into how transcriptional programs are impacted by variation. In addition, our analysis has provided evidence that variants detected at the cellular level can be useful for comparing different cell types in terms of variant impact and burden. The presented method is flexible and can be integrated into analyses of single and bulk-level RNA-sequencing datasets.

## Methods

XCVATR takes aligned BAM-formatted mapped-read files as input. Bulk datasets that contain many BAM files, such as those with one BAM file per sample, can also be input to XCVATR. XCVATR makes use of SAMtools to process BAM files and depends on a SAMtools installation. XCVATR also depends on an R installation for visualization of results.

### Quantification and distance matrix generation

As the first step, reads for each gene from each cell (or sample) are counted. XCVATR makes extensive use of the “CB:Z:” tag that is assigned by the CellRanger software suite for assigning reads to different cells. This tag is also used internally by XCVATR to process bulk samples, so that the same implementation can handle single-cell and bulk samples. The count matrix is used for generating an embedding of the cells in lower dimensions that will be used for detection of variant clumps on the embeddings. The count matrix can also be used for building a cell-to-cell distance matrix. Currently, XCVATR is able to generate t-SNE- and UMAP [62]-based embeddings of cells in lower dimensions and can use the distances from these embeddings. For single-cell datasets, XCVATR can also use the SEURAT package to generate the t-SNE/PCA/UMAP-based embeddings, and for bulk samples, it utilizes the “rtsne” function in R.

### Variant detection and annotation

XCVATR generates coverage pileups for each nucleotide at each position to detect variants. This step can be optionally skipped if there is an existing variant call set (i.e.*,* a VCF file) generated by another pipeline, such as GATK [34] or Mutect [35]. Users can provide these as input and skip the variant detection step.

### SNV detection

To identify SNVs, reads are first deduplicated (by default using SAMtools) and mapping-quality filtered reads (mapping quality > 30, by default) from all cells are used to generate strand-specific pileups at each position on the genome. That is, reads mapping only to the positive and negative strand are used to build the positive-strand and negative-strand pileups, respectively. Next, positions on the pileups are filtered with respect to a minimum alternate AF cutoff and a minimum number of reads that support alternate alleles. XCVATR also filters variants with respect to strand bias, using the strand-specific pileups. Stranded RNA-seq signals must be analyzed without the strand bias parameter. By default, XCVATR uses a total read coverage of at least 10 and a minimum of four mutation-supporting alternative reads, with a minimum alternative AF of 0.2. This filtering-based variant calling method is similar to that used by the VarScan suite of variant callers. For single-cell datasets, XCVATR identifies variants from a BAM file containing the reads from all cells, whereas for bulk datasets, XCVATR analyzes each file/sample separately.

### Indel detection

XCVATR scans all reads and identifies those that support indels by inspecting CIGAR strings in each read. Indel-containing reads are clustered to identify insertions and deletions and then filtered with respect to mapQ, AF, and strand bias.

### Variant annotation

XCVATR uses a GTF- or GFF-formatted annotation file and annotates variants based on their impact on protein-coding sequences. Currently, XCVATR does not annotate impact of non-coding elements. XCVATR maps variants onto the transcripts that are specified in the GTF/GFF files. Variants are then classified with respect to their location: coding sequence (CDS), splice site (two nucleotides at the 5′ and 3′ ends of introns), start/stop codons. CDS mutations are mapped onto the gene sequence, and impacts on coding are evaluated. For SNVs, variants are classified as synonymous, non-synonymous, splice-altering, or start/stop loss; these are the most important impacts that can be assessed in XCVATR. For indels, variants are classified as frameshift/in-frame (CDS-overlapping length in a multiple of three indicates in-frame), splice-altering, or start/stop loss. We have performed extensive comparison of XCVATR annotations to annotations provided by VEP and observed very high concordance.

### Variant filtering

The mutations that are detected in previous step are filtered to decrease computational burden. XCVATR filters the variants by:

1. dbSNP allele frequency lower than 1%
2. Variant annotation belonging to one of the damaging classes, which are one of the classes
  - non-synonymous,
  - nonsense (Early stop codon anywhere on the transcript),
  - splice loss by acceptor/donor variants,
  - inframe/frame-shift indels,
  - stop-disruption.

### Allele counting

For SNVs and small indels, XCVATR counts the number of reads that support the corresponding variants from the BAM file. The “CB:Z” tag is used to assign reads to cells. For each variant, XCVATR tracks the number of reads supporting the alternate and reference alleles.

CNV variants are first separated into amplifications and deletions (Fig. 1b). For large-scale (chromosome arm length) CNVs, XCVATR makes use of the call matrix and generates a count matrix (similar to the allele count matrix) that indicates the existence of a deletion/amplification for the each of the 44 chromosomal arms. For segment-level CNVs, breakpoints of all CNV segments from all cells are pooled and sorted. The sorted breakpoint list is used to define the minimal set of CNV segments whose coordinates do not overlap with breakpoints (Fig. 1b), and this minimal set of segments is used to build a binary count matrix similar to the large-scale matrix.

### Gene-level summarization of SNVs/Indels

Following allele counting, XCVATR iterates over each cell and each gene and assigns allele counts from mutations with the highest impact to each gene, as follows:

$${sAF}_{g_l, cel{l}_i}=\underset{\left\{\forall \operatorname{var}\in {\textrm{V}}_{{\textrm{g}}_{\textrm{l}}}\right\}}{\max}\left({vAF}_{cel{l}_i}^{va{r}_k}\ |\ {vAF}_{cel{l}_i}^{va{r}_k}>0;\forall va{r}_m\in {V}_{g_l}, impact\left( va{r}_k\right)> impact\left( va{r}_m\right)\right)$$

where the summarized AF for gene *g*_*l*_ is set as the AF of the variant *k* whose impact is highest among the variants that overlap with *g*_*l*_, which are denoted by ${V}_{g_l}$. This gene-level summarization accounts for the positioning of the variants and removes some of the information. It is worth noting that summarization is an optional step, as clump detection can be performed at the variant level.

### COSMIC variant frequency assignment to cells

XCVATR can also be used to summarize multiple mutations to analyze different variant-sets. We used COSMIC catalog of cancer variants as it is most prevalent to cancer genomics. XCVATR first overlaps all the detected mutations with COSMIC catalog of variants. Next, for each cell, XCVATR calculates the fraction of COSMIC variants that harbor expression above a certain cutoff. Finally, each cell is assigned an “expressed COSMIC variant fraction” that is calculated as the total number of COSMIC variants expressed in the cell divided by all of the COSMIC mutations in the sample.

### Smoothing scale selection on the embedding

Each scale in the multiscale clump analysis defines the neighborhood around a cell and is used to determine the size of the Gaussian filter around the center cell. Scales are selected to match the scale of the distance metric (or the embedding coordinates). For each cell, XCVATR identifies the *N*_*ν*_ cells that are closest neighbors and calculates neighborhood sizes that cover between 1% (or 10 cells, if lower) and 10% of the cells in the sample (*N*_*cells*_), as follows

$$\left(0.1\times {N}_{cells}\right)>{N}_{\nu }>\max \left(0.01\times {N}_{cells},10\right)$$

It then computes each neighborhood radius, which is defined as the distance from the furthest cell to the current center cell. The minimum and maximum scales (*σ*_*min*_, *σ*_*max*_) are defined as the median of the neighborhood radii of all cells computed at the minimum and maximum neighborhood sizes *N*_*ν*_ defined above. These scales are used for smoothing the AFs and identifying the variant clumps.

### Variant clump candidate selection

Given a smoothing scale *σ*_*a*_ at the scale *a*, XCVATR computes a smoothed AF value for each cell using Gaussian filtering around the cell, as follows:

$${\overline{\phi}}_i^{(a)}=\frac{1}{\left|{N}_{\nu}^{(a)}(i)\right|}\times {\sum}_{k\in {N}_{\nu}^{(a)}(i)\backslash i}{\phi}_j\times \exp \left(-\frac{d_{i,j}^2}{\sigma_a^2}\right)$$

where *ϕ*_*j*_ denotes that alternative AF of the variant in the *j*^*th*^ cell (1 > *ϕ*_*j*_ > 0), *d*_*i*, *j*_ denotes the distance between the *i*^*th*^ and *j*^*th*^ cells in the sample, and ${N}_{\nu}^{(a)}(i)$ indicates the set of indices for the cells that are in the vicinity of the *i*^*th*^ cell for the scale *a*. XCVATR then identifies candidate clump centers as the cells whose smoothed AF is a local maximum among its neighbors (Fig. 1c, step 2), as follows:

$${C}_a=\left\{i\left|\forall j\in {N}_{\nu}^{(a)}(i):{\overline{\phi}}_i^{(a)}>{\overline{\phi}}_j^{(a)}\right.\right\}$$

where *C*_*a*_ denotes the indices of cells that are clump centers.

### Specification of position and size of clumps

The scale at which a clump is discovered (i.e.*, σ*_*a*_) is used to define the initial size of each clump.

### Variant clump evaluation by RD-aware permutation

For clump center candidate cell *C*_*a*_ (*a*^*th*^ scale), XCVATR permutes the AFs *Κ* times and computes the smoothed AF for every candidate. XCVATR then computes a *z*-score that is used to rank the clumps, as follows:

$${Z}_{\phi }=\frac{{\overline{\phi}}_i^{(a)}-{\mu}_{\phi }}{\delta_{\phi }}$$

AFs of cells whose coverage is greater than *τ* are used for shuffling, whereas other cells are not shuffled and are not used for the permutation test. This allows XCVATR to control for RD biases.

### Read-level enrichment of alternate allele expression in clumps

XCVATR first counts the total number of alternative and reference reads in all cells. These represent the baseline (bulk) read-level alternate AF. For each clump, the total alternate allele-supporting reads and total reads are counted. At scale *a*, for the *b*^*th*^ clump, these are used to compute the read-level modified binomial *P*-value, as follows:

$$Bin\left({n}_{alt}^{\left(a;b\right)},{n}_{ref}^{\left(a;b\right)};p=A{F}_{alt}^{(bulk)}\right)={\sum}_{\begin{array}{c}{n}_{alt}^{\left(a;b\right)}>r>1;\\ {}t=\left({n}_{ref}^{\left(a;b\right)}+{n}_{alt}^{\left(a;b\right)}-r\right)\end{array}}\left(\begin{array}{c}{n}_{ref}^{\left(a;b\right)}+{n}_{alt}^{\left(a;b\right)}\\ {}i\end{array}\right){p}^r\times {\left(1-p\right)}^t$$

where ${n}_{ref}^{\left(a;b\right)}$ and ${n}_{alt}^{\left(a;b\right)}$ denote the number of reads that support alternate and the reference alleles, respectively. For the corresponding variant in the *b*^*th*^ clump identified in scale *a*:

$${n}_{ref}^{\left(a;b\right)}={\sum}_{j\in {N}_v^{\left(a;b\right)}}{n}_{ref}(j),{n}_{alt}^{\left(a;b\right)}={\sum}_{j\in {N}_v^{\left(a;b\right)}}{n}_{alt}(j)$$

where ${N}_v^{\left(a;b\right)}$ denotes the neighborhood of the cell at the center of clump *b* at the scale *a*, and *n*_*ref*_(*j*) indicates the number of reference alleles in cell *j*. In the above equation, the flip probability is chosen as $p=A{F}_{bulk}=\frac{n_{alt}^{(bulk)}}{n_{alt}^{(bulk)}+{n}_{ref}^{(bulk)}}$, which represents the alternative AF of the variant in the whole bulk sample. The binomial *P*-value estimates enrichment of the alternative allele-supporting reads in the clump *b* when compared to randomly assigning reads to all cells with probability *p* = *AF*_*bulk*_.

### Cell-level enrichment of alternate allele expression in the clumps

At scale *a*, XCVATR counts the cells in clump *b* with alternative AFs above *η* (By default 0.5). Next, XCVATR counts the number of cells in the whole sample for which the alternative AF is above *η*. These values are used to compute a significance of the enrichment of alternative alleles at cell level using Fisher’s exact test, as follows:

$$F{E}^{\left(a;b\right)}= FE\left({c}_{\eta}^{\left(a;b\right)}(j),{c}_0^{\left(a;b\right)}(j);{c}_{\eta}^{(bulk)},{c}_0^{(bulk)}\right)$$

where ${c}_{\eta}^{\left(a;b\right)}(j)$ indicates the number of cells in clump *b* at scale *a*, for which AF exceeds *η*:

$${c}_{\eta}^{\left(a;b\right)}(j)={\sum}_jI\left({\phi}_j^{\left(a;b\right)}>\eta \right).$$

and ${c}_{\eta}^{(bulk)}$ denotes the number of cells among all the cells (i.e.*,* bulk) for which the AF exceeds *η*. The read-level and cell-level enrichment estimates are used to filter out clumps that exhibit low levels of enrichment in comparison to the bulk sample at read and cell (or sample) level.

### Filtering of the clumps detected by XCVATR

We recommend downstream filtering of the clumps identified by XCVATR and further visualization to make sure relevant clumps are properly assessed. We suggest using the assigned clump z-score (greater than 2), number of cells that harbor the mutation (greater than 10), and cell-level and read-level significance *p*-values (Smaller than 5%). The users can choose to adjust these filters since different datasets may have higher or lower coverage of mutations and some cell populations may be rare.

### Visualization

XCVATR makes use of R scripts to visualize clumps on the embedding coordinates. The visualization utilities are implemented in R and directly make use of the data generated by XCVATR.

### Clump simulation for testing clump detection accuracy

Clump simulation starts by selection of a random cell among the 1170 cells in BT_S2 sample of Darmanis et al. dataset [43]. Given the simulated clump center cell, we then shuffle the AFs of all other cells based on their distance and AFs. This shuffling is performed by starting from the closest neighbor of the clump center, randomly sampling a cell to this position (without replacement), then moving to the next neighbor. It is repeated until no cells are left without an AF. To simulate a clump shape, we used two parameters while sampling: “distance weight”, *δ*, and “AF weight”, *α*. Given that we are sampling the AF for the cell whose distance to the clump center is *d*_*i*_, the weight of the cell is assigned as:

$${w}_i= pow\left({\phi}_i,\alpha \cdot \exp \left(-1\cdot \delta \cdot {d}_i\right)\right),$$

where *w*_*i*_ denotes the weight for the *i*^*th*^ cell, and *pow*(*a*, *b*) = *a*^*b*^. Sampling weights are proportional to the probability of selecting the cells, and at long distances (i.e., large *d*_*i*_), the sampling weights of all cells (regardless of *ϕ*_*i*_) are almost equal to each other (i.e., *w*_*i*_ = 1). When the distance to the simulated clump center is small, sampling weights for cells with higher AFs are higher compared to sampling weights for cells with lower AFs, such that shuffling “attracts” high AFs closer to the clump center. After sampling weights are assigned to all cells, a cell is randomly selected, whereby the probability of the selected cell *i* is proportional to *w*_*i*_. The selected cell’s alternative and reference allele counts are copied to the current cell-of-focus (at distance *d*_*i*_), and the selected cell is removed from the list of cells to ensure the same cell is not assigned twice. This sampling process is continued until all cells are assigned with an AF.

### CNV calling by CaSpER

The CaSpER [11] algorithm was used to detect CNVs. For each dataset, normal cells (e.g., immune cells) are used as controls to identify somatic CNVs in tumor cells.

### Single-cell meningioma analysis

Postal and frontal meningioma samples were sequenced at the MD Anderson Epigenetics Core. CellRanger was run with default parameters, and we then used default parameters in the SEURAT package to filter out dead cells and doublets and generate the UMAP embedding of tumor cells.

## Supplementary Information

**Additional file 1: Supplementary Table 1.** The list of deletion clumps detected by XCVATR in Darmanis et al. dataset. We report the large-scale deletions, the reported z-score, number of cells that support the deletion, and the scale at which the clump is detected. **Supplementary Table 2.** The list of amplification clumps detected by XCVATR in Darmanis et al. dataset. We report the large-scale amplifications, the reported z-score, number of cells that support the amplification, and the scale at which the clump is detected. **Supplementary Table 3.** The gene-level variant clumps detected in Darmanis et al. dataset. The impacted genes, z-score, number of cells with gene mutation, and the clump scale is reported. **Supplementary Figure 1.** Left panel shows the embedding coordinates for each chromosome that is used by XCVATR to search for potential amplification clumps. Color scale indicates the smoothed allele frequency of the amplification in the vicinity of the candidate clump center. Chromosomes with strong clump candidate centers are depicted with red rectangles. Right panel shows the distribution of the smoothed allele frequency around the candidate clump centers at each chromosome. **Supplementary Figure 2.** Left panel shows the embedding coordinates for each chromosome that is used by XCVATR to search for potential deletion clumps. Color scale indicates the smoothed allele frequency of the amplification in the vicinity of the candidate clump center. Right panel shows the distribution of the smoothed allele frequency around the candidate clump centers at each chromosome. **Supplementary Figure 3.** Estimated drop-out rate for each chromosome in meningioma samples. X-axis shows the chromosome and y-axis shows the estimate of drop-out rates.

## Data Availability

The scRNA-seq datasets from Darmanis et al. [43] are available from the Gene Expression Omnibus (GEO) database with the accession number: GSE84465. 160 sample Meningioma RNA-seq dataset is downloaded from Patel et al. [42] The 10X single cell sequencing data and the sample information for 2 meningioma tumors are deposited to GEO database under accession identifier GSE213544.

## Abbreviations

XCVATR: Expressed Clusters of Variant Alleles in Transcriptome Profiles
GATK: Genome analysis toolkit
dbSNP: The single nucleotide polymorphism database
GTF/GFF: Gene transfer format / General feature format
COSMIC: Catalog of somatic mutations in cancer
t-SNE: t-distributed stochastic neighbor embedding
UMAP: Uniform manifold approximation and projection
PCA: Principal component analysis
eQTL: Expression Quantitative Trait Locus
CDS: Coding sequence
RD: Read depth
AF: Allele frequency
MAF: Minor allele frequency
RNA-seq: RNA sequencing
scRNA-seq: Single cell RNA sequencing
CNV: Copy number variant
SNV: Single nucleotide variant
Indel: Insertion/deletion
BAM: Binary alignment map
SAM: Sequence alignment map
HMM: Hidden Markov model
mapQ: Mapping quality
