# Supplementary material for: XCVATR: detection and characterization of variant impact on the Embeddings of single -cell and bulk RNA-sequencing samples
Source: BMC Genomics. 2022 Dec 20;23:841. doi: 10.1186/s12864-022-09004-7 (PMC9764736; doi:10.1186/s12864-022-09004-7)
Supplement: Supplementary file 1 — Additional file 1: Supplementary Table 1. The list of deletion clumps detected by XCVATR in Darmanis et al. dataset. We report the large-scale deletions, the reported z-score, number of cells that support the deletion, and the scale at which the clump is detected. Supplementary Table 2. The list of amplification clumps detected by XCVATR in Darmanis et al. dataset. We report the large-scale amplifications, the reported z-score, number of cells that support the amplification, and the scale at which the clump is detected. Supplementary Table 3. The gene-level variant clumps detected in Darmanis et al. dataset. The impacted genes, z-score, number of cells with gene mutation, and the clump scale is reported. Supplementary Figure 1. Left panel shows the embedding coordinates for each chromosome that is used by XCVATR to search for potential amplification clumps. Color scale indicates the smoothed allele frequency of the amplification in the vicinity of the candidate clump center. Chromosomes with strong clump candidate centers are depicted with red rectangles. Right panel shows the distribution of the smoothed allele frequency around the candidate clump centers at each chromosome. Supplementary Figure 2. Left panel shows the embedding coordinates for each chromosome that is used by XCVATR to search for potential deletion clumps. Color scale indicates the smoothed allele frequency of the amplification in the vicinity of the candidate clump center. Right panel shows the distribution of the smoothed allele frequency around the candidate clump centers at each chromosome. Supplementary Figure 3. Estimated drop-out rate for each chromosome in meningioma samples. X-axis shows the chromosome and y-axis shows the estimate of drop-out rates. [file 12864_2022_9004_MOESM1_ESM.pdf]

Supplementary Information for *"XCVATR: Detection and Characterization of Variant Impact on the Embeddings of Single -Cell and Bulk RNA-Sequencing Samples"*

Arif Harmanci[1,*,+] , Akdes Serin Harmanci[2,*], Tiemo J. Klisch[4,5], Akash J. Patel[2,3,4,+]

1 University of Texas Health Science Center, School of Biomedical Informatics, Center for Precision Health

2 Department of Neurosurgery, Baylor College of Medicine, Houston, TX, 77030

3 Department of Otolaryngology – Head and Neck Surgery, Baylor College of Medicine, Houston, TX 77030

4 Jan and Dan Duncan Neurological Research Institute, Texas Children's Hospital, Houston, TX 77030

5 Department of Molecular and Human Genetics, Baylor College of Medicine, Houston, TX 77030

**These authors contributed equally.*

[+]*Corresponding authors*

Introduction

We include supplementary discussion Supplementary Tables and Figures.

Significance and Downstream Analysis of Variant Clumps

As we discussed in our manuscript, definition and estimation of clumps in multidimensional embedding domain and higher dimensions is not easy and we consider it a future direction that should be studied further. There are similar problems in bioinformatics and image analysis. For example, "peak calling" in functional genomics data such as ChIP-Seq and ATAC-Seq can be thought of as detection of clumps in a 1-dimensional read-depth data to identify the genomic coordinates with significant increase in the read coverage. Each peak corresponds to a genomic range that indicates the potential position of a DNA-protein interaction (or histone modification) is rather vague. The peak calling algorithms usually generate a very large number of potential peaks and downstream analysis (such as motif finding) is generally required to focus on the most significant peaks. This is similar to our clump detection scenario: We recommend scoring the clumps and performing downstream analysis by manual assessment of the visualizations and programmatic extraction of cells and analysis of cell-type-specific markers etc.

Secondly, blob detection in image analysis represents a similar problem, perhaps more similar in a 2-dimensional context. In blob detection, the portions of the image that show uniform pattern are detected as single entities called "blobs", which is conceptually similar to the clumps. A number of blob detection methods are based on multiscale decomposition of images to identify the blobs at different scales and reconciling the blobs identified in each scale to generate the "final blob calls". There is no clear definition

of a blob in this framework. In image processing aspect, this makes sense since at different scales, one can identify different textures and features that reveal different information. For example, take the image of a human face: At the large scales, the blobs correspond to nose, eyes, forehead, and ears. At a fine scale, finer details are revealed such as the eye's pupil and iris will have their own blobs within the larger "eye blob" identified in the larger scale. Thus, each scale can provide a specific information about the blobs or clumps, in our specific case.

XCVATR utilizes a similar multiscale approach to systematically identify and score clumps. Similar to blob detection problem in image processing, the clumps can exhibit a multiscale structure that can extend to different scales. This is a reasonable expectation since different clumps can potentially correspond to different clones of cells that exhibit similar global expression profiles and therefore are close to each other in the embedding domain. In Fig. 3b, although we highlighted two clumps, there is also a clump that combines them together shown in the light blue color. It may therefore be necessary for the users to perform downstream analysis to characterize these clumps further.

To ensure that XCVATR focuses on the correct set of clumps (and also for computational efficiency), XCVATR detects a candidate set of clump "centers" by identifying the local maxima of the smoothed signal at different smoothing scales (Methods Section). Of note, this step is similar to the Gaussian and wavelet multiscale decomposition analysis of images to identify blobs(Moulin 2009). This step can be performed much quicker than the scoring of the clumps since both smoothing and local maxima detection can be performed very quickly. To illustrate the candidate clumps over all other chromosomes, we now included Supplementary Figures 1 and 2 that shows the evaluated clump centers and the distribution of smoothed allele frequencies over all chromosomal arms. The strongest clump centers for both amplifications and deletions that XCVATR identifies are most relevant to glioma tumors. As an example, Fig. 3b shows 17q for illustration. We added a new table that lists the other clumps that XCVATR identifies for chromosomes 4, 12, and 13 (Supplementary Table 1). These chromosomes are frequently altered in Glioma samples. For amplifications, XCVATR's filtered clumps are found on chromosomes 7, 20, and 21 (Supplementary Table 2).

Existing Metrics that Define Spatial Correlation. Other metrics such as Moran's-I correlation statistic(Moran 1950), which is used extensively in ecology and spatial data analysis could be used in this context. However, there are specific challenges to using these existing metrics in the embedding coordinates. First, the embedding coordinates are non-uniform, unlike 2D datasets such as an image or spatial RNA sequencing (i.e., Slide-Seq or Visium), where the measurements are made on a uniform grid where each measurement corresponds to a point in the space while the embedding places each data point in a non-uniform space. We believe it is therefore more appropriate to analyze the embedding datasets using network or graph-based methods that SEURAT uses and similarly used in our method to first build a nearest-neighbor map.

Downstream Analysis of Clumps. An important component of clump analysis is the manual assessment of the results. We foresee that a qualitative or programmatic correlation of the clumps with other features (e.g. cell phenotypes, cell types, or marker gene expression levels) and further analysis of the clumps is necessary to characterize the clumps better. This can be performed manually by overlaying the clump coordinates to other covariates such as gene expression plots. This can also be performed programmatically by extracting the cell identifiers in the vicinity of the identified clump from the embedding file and selecting these cells from the allelic count file or gene expression counts from

CellRanger. This can be performed very easily in R by loading the expression matrix using Seurat and selecting the cells around the clump that are extracted in the previous step. After this, differential expression, co-expression, and pathway enrichment analysis can be performed using existing tools. We have thought of the possibility of adding a pathway-specific analysis to XCVATR but concluded that it would be overlapping to a large extent with existing tools and would not provide much additional insight.

Impact of Drop-out Events

Dropouts have complex effects on detection and analysis of copy number variants. Firstly, the drop-outs can be confounded (or correlated) by a large-scale deletion event because the genes on a deleted copy of the chromosome will more likely to have zero counts as they can be sampled less from the pool of cDNA molecules. Secondly the CNV calling pipelines will be adversely impacted from this since they rely on existence of a continuous set of genes on a chromosome to be able to segment the gene expression profile over consecutive genes.

Furthermore, previous studies do not have conclusive explanation or treatment of dropout events(Kim, Zhou, and Chen 2020). On one hand, dropouts have a technical component where dropout is caused by stochastic nature of cDNA sampling(Ran et al. 2020). On the other hand, number of studies have shown real biological dropouts where different cell types can be identified by analysis of only drop-out patterns without any regard to the actual expression levels of the cells (Qiu, 2020, Nature Communications). This is important in tumor samples to analyze heterogeneity. We and others have recently been working on the hypothesis that meningioma may exhibit intratumor heterogeneity between 3 classes (namely A, B, and C) that have distinct CNV profiles in the genomes(Bayley et al. 2022). We therefore believe the analyses and filtering of the cells should be performed carefully to be able to test these hypotheses.

For these reasons, the dropouts are not explicitly filtered out except for extremely lowly represented genes: SEURAT filters out cells that have less than 200 "features" in them with non-zero counts. It is also not clear how a systematic cutoff could be set for removing cells or genes (or features) with high dropout rates as these can potentially be indicative of rare cell types. One other option is to impute the dropouts using approaches such as NMF or nearest neighbor-based Markov field smoothing methods such as MAGIC(van Dijk et al. 2018). This may, however, implicitly smooth out or introduce copy number variants into cells that may not otherwise harbor these variants. It is not clear how well imputation methods are suited for cancer samples with large scale events. This may also have an adverse effect on clump analysis because XCVATR also performs a separate smoothing on the variant calls using nearest-neighbors and it may amplify the biases that are introduced by the imputation algorithm. We therefore refrain from using imputed allele frequencies or read counts and use raw alternate allele counts.

In our analysis, we focused on a specific set of chromosomes that have concordant genotyping array calls on the bulk samples. We have now added an estimated level of dropout in Frontal and Postal samples. We estimate around 86% overall dropout rate over whole genome. We next stratified the dropout estimates per chromosome and found that the top 6 chromosomes that exhibit highest level of dropout rates are the chromosomes that harbor large scale deletions that are well-known in meningioma (Fig. S3). These are chromosomes 22, 10, 18, 14, and 1. We also found chromosome 11 has high dropout rate, which was also found in the genotyping array.

We would like to point out that these events are driver events that are commonly found in Meningioma's B and C classes and they are expected to occur in very high frequency among tumor cells, we therefore

expect a to observe a high fraction of these deletions. The reason why we focused on these deletions is to ask whether XCVATR identifies any intratumor heterogeneity at the embedding of the cells using global expression profiles. From our results, there is some evidence, there is a certain amount of heterogeneity which may indicate that tumor cells could be further subclassified within a tumor. Another possible explanation of this could easily be that there are normal cells that are mixed into tumor sample.

Deleted Chromosomal Arm	Clump Z-Score	Number of Cells Supporting Deletion	Identified Scale (Inverse Diameter)
17q	38.6667	122	0.0056180000
17q	38.0000	122	0.0189620000
17q	38.0000	122	0.0126420000
17q	37.3333	122	0.0084280000
22q	36.3333	143	0.0084280000
17p	36.0000	114	0.0056180000
17p	35.6667	114	0.0126420000
13q	35.3333	151	0.0037460000
17q	35.2500	107	0.0426650000
17p	34.6667	114	0.0084280000
22q	33.3333	144	0.0037460000
17q	33.3333	123	0.0037460000
10q	33.3333	148	0.0037460000
17q	32.5000	116	0.0284430000
17p	31.7500	102	0.0426650000
22q	31.5000	112	0.0426650000
17p	31.3333	116	0.0037460000
10p	31.0000	141	0.0037460000
13q	30.7500	151	0.0056180000
22q	30.0000	133	0.0284430000
22q	29.5000	144	0.0056180000
13q	29.5000	147	0.0126420000
13q	29.5000	151	0.0084280000
10q	29.2500	132	0.0284430000
10q	28.7500	147	0.0056180000
13q	27.4000	122	0.0426650000
22q	27.2500	140	0.0126420000
10q	27.0000	147	0.0084280000
10q	26.7500	143	0.0126420000
10p	26.7500	125	0.0284430000
10p	26.5000	140	0.0056180000
10p	25.2500	140	0.0084280000
10p	25.0000	136	0.0126420000
10q	24.2000	111	0.0426650000
4q	23.2500	72	0.0426650000
4q	23.2500	82	0.0284430000
10p	23.2000	108	0.0426650000
13q	14.3333	68	0.0426650000

Supplementary Table 1. The list of deletion clumps detected by XCVATR in Darmanis et al. dataset. We report the large-scale deletions, the reported z-score, number of cells that support the deletion, and the scale at which the clump is detected.

Amplified Chromosomal Arm	***Clump Z-Score***	***Number of Cells Supporting Amplification***	***Identified Scale (Inverse Diameter)***
7q	27.0000	240	0.0037460000
7q	25.2500	230	0.0084280000
7q	23.5000	200	0.0126420000
20q	21.7143	23	0.0126420000
7q	21.4000	231	0.0056180000
20q	20.7143	22	0.0189620000
7q	20.2000	144	0.0189620000
7q	18.8333	131	0.0284430000
20q	17.4286	22	0.0084280000
7q	16.5714	99	0.0426650000
7q	15.7778	52	0.0189620000
7q	15.5000	53	0.0126420000
21q	15.0000	37	0.0189620000
7q	14.6471	53	0.0084280000
7q	14.2857	67	0.0037460000
7q	14.0000	56	0.0056180000
7q	8.4000	56	0.0426650000
7p	7.7692	22	0.0284430000
7p	6.7857	22	0.0084280000
7p	6.6000	21	0.0126420000

Supplementary Table 2. The list of amplification clumps detected by XCVATR in Darmanis et al. dataset. We report the large-scale amplifications, the reported z-score, number of cells that support the amplification, and the scale at which the clump is detected.

Gene	Clump Z-Score	Number of Cells Supporting Gene Mutation	Identified Scale (Inverse Diameter)
TCTN3	4.2778	44	0.0117800000
GSTO1	3.9111	32	0.0117800000
TCTN3	3.8824	42	0.0176700000
TCTN3	3.7500	37	0.0265050000
MTG1	3.5676	38	0.0117800000
TP53	3.4898	17	0.0117800000
TCTN3	3.3684	32	0.0397570000
MTG1	3.1026	35	0.0176700000
DST	2.9333	19	0.0117800000
MTG1	2.8684	33	0.0265050000
TCTN3	2.6923	25	0.0894540000
DST	2.6800	14	0.0176700000
TP53	2.6615	12	0.0176700000
TP53	2.5738	11	0.0265050000
DST	2.4286	13	0.0265050000
MTG1	2.3542	25	0.0397570000
MSANTD3	2.2653	26	0.0596360000
MSANTD3	2.2200	21	0.1341800000
MTG1	2.2157	20	0.0596360000
DST	2.1364	11	0.0397570000

Supplementary Table 3. The gene-level variant clumps detected in Darmanis et al. dataset. The impacted genes, z-score, number of cells with gene mutation, and the clump scale is reported.

Supplementary Figure 1

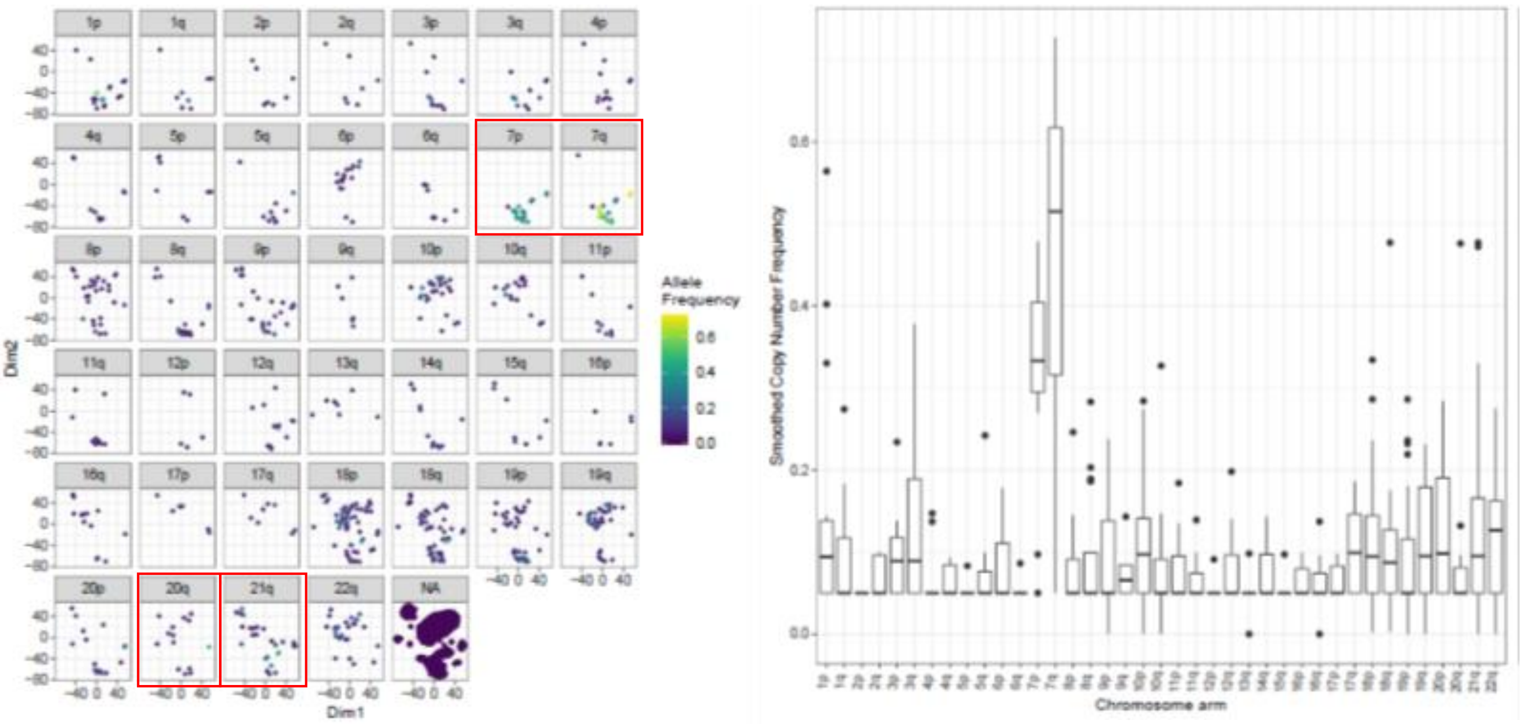

Supplementary Figure 1. Left panel shows the embedding coordinates for each chromosome that is used by XCVATR to search for potential amplification clumps. Color scale indicates the smoothed allele frequency of the amplification in the vicinity of the candidate clump center. Chromosomes with strong clump candidate centers are depicted with red rectangles. Right panel shows the distribution of the smoothed allele frequency around the candidate clump centers at each chromosome.

Supplementary Figure 2

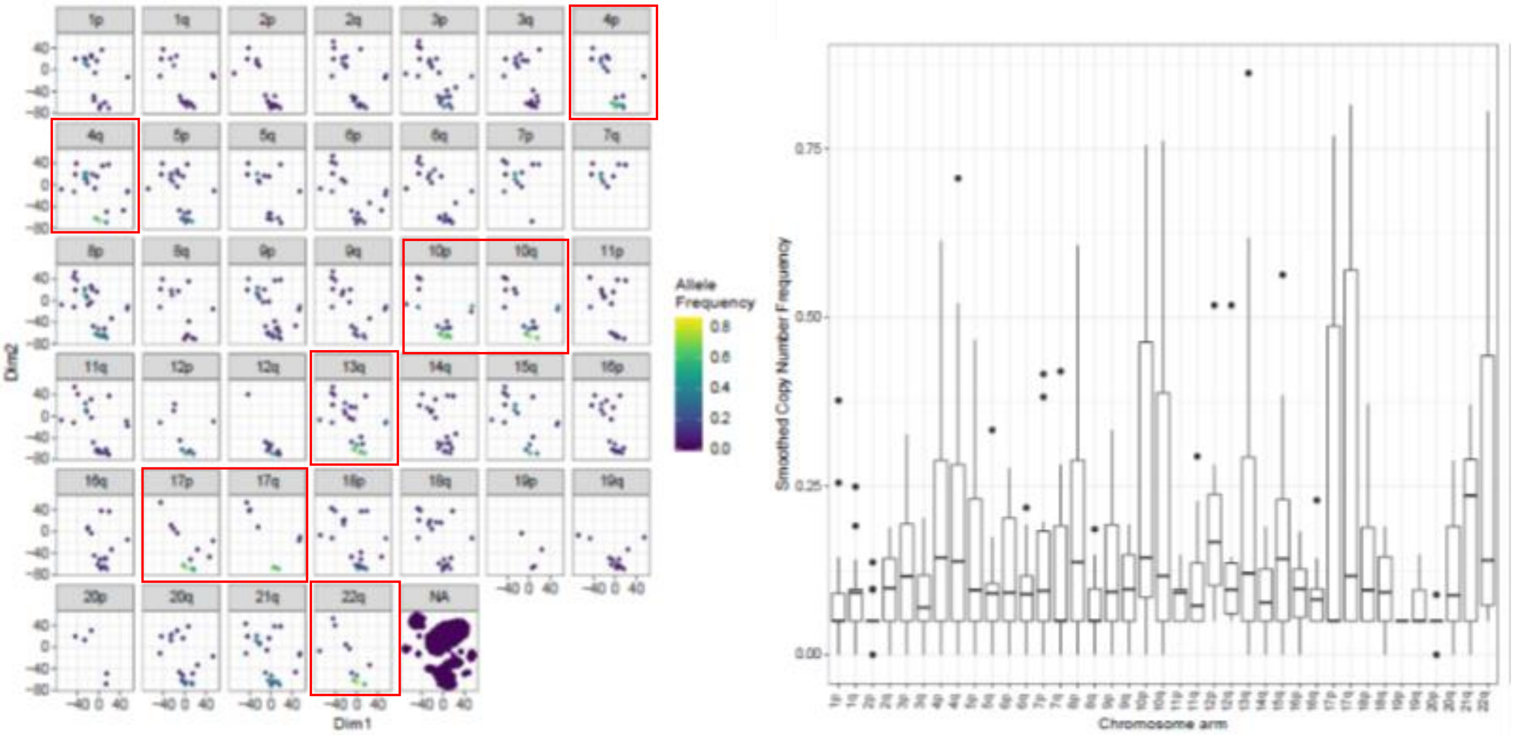

Supplementary Figure 2. Left panel shows the embedding coordinates for each chromosome that is used by XCVATR to search for potential deletion clumps. Color scale indicates the smoothed allele frequency of the amplification in the vicinity of the candidate clump center. Right panel shows the distribution of the smoothed allele frequency around the candidate clump centers at each chromosome.

Supplementary Figure 3

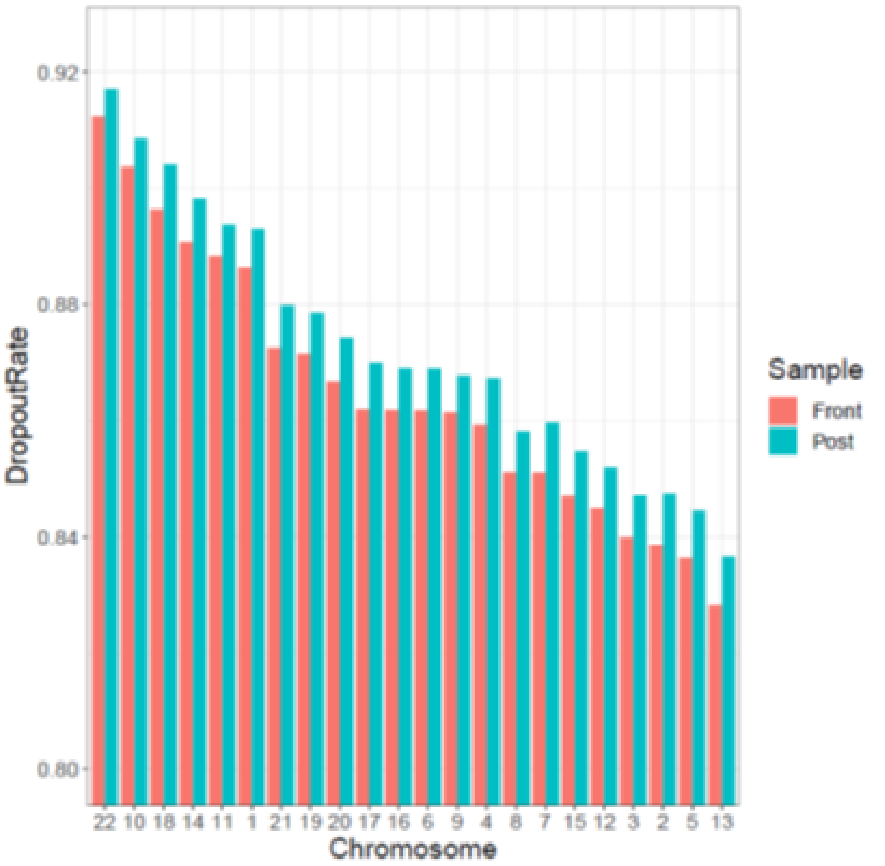

Supplementary Figure 3. Estimated drop-out rate for each chromosome in meningioma samples. X-axis shows the chromosome and y-axis shows the estimate of drop-out rates.

REFERENCES

Bayley, James C., 5th, Caroline C. Hadley, Arif O. Harmanci, Akdes S. Harmanci, Tiemo J. Klisch, and Akash J. Patel. 2022. “Multiple Approaches Converge on Three Biological Subtypes of Meningioma and Extract New Insights from Published Studies.” *Science Advances* 8 (5): eabm6247.

Dijk, David van, Roshan Sharma, Juozas Nainys, Kristina Yim, Pooja Kathail, Ambrose J. Carr, Cassandra Burdziak, et al. 2018. “Recovering Gene Interactions from Single-Cell Data Using Data Diffusion.” *Cell* 174 (3): 716-729.e27.

Kim, Tae Hyun, Xiang Zhou, and Mengjie Chen. 2020. “Demystifying ‘Drop-Outs’ in Single-Cell UMI Data.” *Genome Biology* 21 (1): 196.

Moran, P. A. P. 1950. “Notes on Continuous Stochastic Phenomena.” *Biometrika* 37 (1–2): 17–23.

Moulin, Pierre. 2009. “Multiscale Image Decompositions and Wavelets.” In *The Essential Guide to Image Processing*, 123–42. Elsevier.

Ran, Di, Shanshan Zhang, Nicholas Lytal, and Lingling An. 2020. “ScDoc: Correcting Drop-out Events in Single-Cell RNA-Seq Data.” *Bioinformatics (Oxford, England)* 36 (15): 4233–39.
